# Asparagine drives immune evasion in bladder cancer via RIG-I stability and type I IFN signaling

**DOI:** 10.1172/JCI186648

**Published:** 2025-02-18

**Authors:** Wenjie Wei, Hongzhao Li, Shuo Tian, Chi Zhang, Junxiao Liu, Wen Tao, Tianwei Cai, Yuhao Dong, Chuang Wang, Dingyi Lu, Yakun Ai, Wanlin Zhang, Hanfeng Wang, Kan Liu, Yang Fan, Yu Gao, Qingbo Huang, Xin Ma, Baojun Wang, Xu Zhang, Yan Huang

**Affiliations:** 1 Department of Urology, The Third Medical Center and; 2 Department of Urology Laboratory, Chinese PLA General Hospital, Beijing, China.; 3 Medical School of PLA, Beijing, China.; 4 State Key Laboratory of Proteomics, Institute of Basic Medical Sciences, National Center of Biomedical Analysis, Beijing, China.; 5 Department of Pathology, The Third Medical Center, Chinese PLA General Hospital, Beijing, China.

**Keywords:** Cell biology, Immunology, Therapeutics, Immunotherapy, Urology

## Abstract

Tumor cells often employ many ways to restrain type I IFN signaling to evade immune surveillance. However, whether cellular amino acid metabolism regulates this process remains unclear, and its effects on antitumor immunity are relatively unexplored. Here, we found that asparagine inhibited IFN-I signaling and promoted immune escape in bladder cancer. Depletion of asparagine synthetase (ASNS) strongly limited in vivo tumor growth in a CD8^+^ T cell–dependent manner and boosted immunotherapy efficacy. Moreover, clinically approved L-asparaginase (ASNase),synergized with anti–PD-1 therapy in suppressing tumor growth. Mechanistically, asparagine can directly bind to RIG-I and facilitate CBL-mediated RIG-I degradation, thereby suppressing IFN signaling and antitumor immune responses. Clinically, tumors with higher ASNS expression show decreased responsiveness to immune checkpoint inhibitor therapy. Together, our findings uncover asparagine as a natural metabolite to modulate RIG-I–mediated IFN-I signaling, providing the basis for developing the combinatorial use of ASNase and anti–PD-1 for bladder cancer.

## Abstract 2

Asparagine directly regulates RIG-I stability and type I IFN signaling to drive immune evasion in bladder tumor.

## Graphical abstract

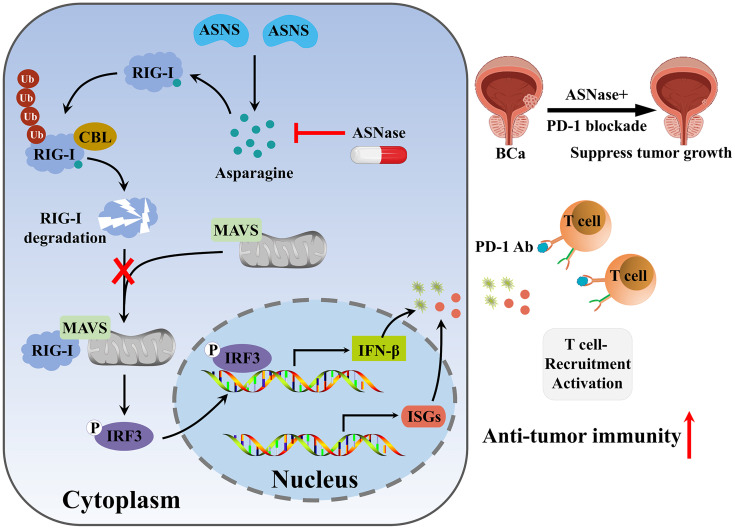

## Introduction

Bladder cancer is one of the most prevalent tumors in the urinary system and is responsible for nearly 165,000 deaths every year worldwide (1). Approximately 25% of patients present with muscle invasive disease and the relative 5-year overall survival rate of advanced-stage bladder cancer is low, with limited therapeutic advances (2, 3). Radical cystectomy and cisplatin-based chemotherapy are recommended for the standard treatment option for muscle-invasive bladder cancer (MIBC) (4). In recent years, immune checkpoint inhibitors (ICIs) have achieved tremendous clinical breakthroughs in cancer immunotherapy (5, 6). Though ICIs have shown durable responses in a subset of patients with bladder cancer, the overall response rate is only approximately 15%–25% (7, 8), which increases the demand for biomarkers of response and effective targeted therapies to enhance ICI therapy.

Emerging evidence indicates that the tumor microenvironment (TME) is a complex system that determines the occurrence of tumor immune responses (9, 10). CD8^+^ T cells play a central role in cancer immunotherapy and elicit antitumor activity by directly recognizing and killing tumor cells (11, 12). Patients with high CD8^+^ T cell infiltration in TME are associated with a survival benefit in several tumor types and better response to immunotherapy (13). Previous studies have reported that IFN-I is essential for activating adaptive immune response and plays a crucial role in CD8^+^ T cell infiltration and immunogenic tumor rejection (14, 15). Given that retinoic acid-inducible gene-I–like (RIG-I–like) receptors (RLRs) are critical for activating the IFN pathway and triggering immunogenic cell death, stimulation of RIG-I or melanoma differentiation–associated gene 5 (MDA5) signaling has emerged as a strategy for antitumor immunity (16, 17). However, tumor cells often employ multiple strategies to evade immunosurveillance by inhibiting RLR-mediated signaling pathway (18, 19). Therefore, a better comprehension of the regulatory mechanism of RLR-mediated IFNs in tumor immunity is of great clinical importance for patients with bladder cancer.

Tremendous efforts have recently been dedicated to the investigation of metabolic reprogramming in the regulation of tumor immunity (20–22). Among them, amino acid metabolism has attracted widespread attention. Asparagine synthetase (ASNS) catalyzes the conversion of aspartate to asparagine in an ATP-dependent reaction. L-asparaginase (ASNase), a drug that deprives asparagine in patients’ plasma, is considered to be a first-line therapy for childhood acute lymphoblastic leukemia (ALL) (23). Accumulating studies have revealed that asparagine is tightly linked to the activation and differentiation of CD8^+^ T cells, thereby affecting antitumoral functionality (24–26). In addition, ASNS is elevated in many cancer types, and increased asparagine contributes to cancer cell survival and metastasis (27–29). Nonetheless, the specific roles of asparagine metabolism on the cancer cell–intrinsic functions and on the regulation of RLR-mediated antitumor immunity have not been explored.

Here, we report that asparagine restriction enhances RIG-I–mediated IFN signaling and potentiates antitumor immunity in bladder cancer. Clinically, ASNS is upregulated and is associated with a poor response to immunotherapy in bladder cancer. Limiting asparagine by knockdown of ASNS or treatment with ASNase increases intratumoral CD8^+^ T cell infiltration and effector function, thus boosting the efficacy of PD-1 blockade. Mechanistically, asparagine can directly facilitate the interaction between E3 ligase CBL and RIG-I, consequently inducing RIG-I degradation to suppress IFN signaling, thereby limiting antitumor immune responses. Our study highlights a role of asparagine in regulating RIG-I stability and connects asparagine metabolism to the IFN-I signaling that modulates antitumor immunity, suggesting that targeting ASNS is a promising approach to enhance immunotherapy for bladder cancer.

## Results

### Asparagine restriction attenuates tumor growth in an immunity-dependent manner.

Previous studies report that ASNS functions as a critical enzyme that catalyzes the biosynthesis of asparagine from aspartate in an ATP-dependent reaction (23). To interrogate the biological functions of ASNS on cancer cells, we knocked down the *Asns* gene in 2 malignant mouse bladder cancer cell lines (MB49 and MBT2) and a human bladder cancer cell line, UMUC3 (Supplemental Figure 1, A and B; supplemental material available online with this article; https://doi.org/10.1172/JCI186648DS1). Loss of ASNS did not alter the in vitro growth rates and migration abilities of tumor cells (Supplemental Figure 1, C–F). Consistent with this, we found that asparagine also had no obvious difference on the proliferation abilities of bladder cancer cells compared with the control cells (Supplemental Figure 1G).

**Figure 1:**
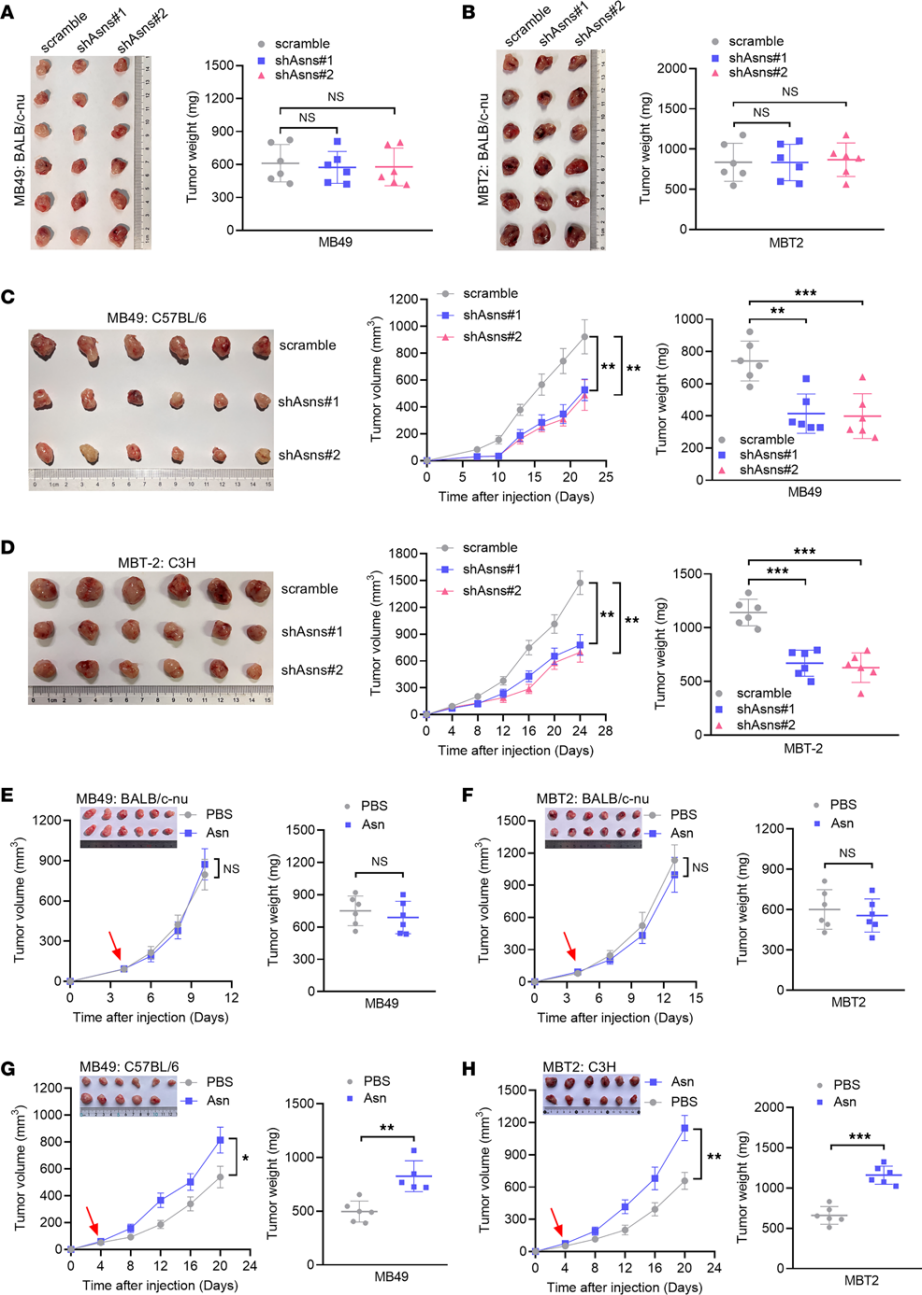
Asparagine restriction attenuates tumor growth in syngeneic mice. (**A**) Tumor image and tumor weight of immunodeficient nude mice (*n* = 6) injected subcutaneously with scramble or shAsns MB49 cells. (**B**) Tumor image and tumor weight of immunodeficient nude mice (*n* = 6) injected subcutaneously with scramble or shAsns MBT2 cells. (**C**) Tumor growth curves and tumor weight of immunocompetent C57BL/6 mice (*n* = 6) injected subcutaneously with scramble or shAsns MB49 cells. (**D**) Tumor growth curves and tumor weight of immunocompetent C3H mice (*n* = 6) injected subcutaneously with scramble or shAsns MBT2 cells. (**E**) Tumor growth curves and tumor weight of immunodeficient nude mice (*n* = 6) injected subcutaneously with MB49 cells administrated with PBS or Asn. (**F**) Tumor growth curves and tumor weight of immunodeficient nude mice (*n* = 6) injected subcutaneously with MBT2 cells administrated with PBS or Asn. (**G**) Tumor growth curves and tumor weight of immunocompetent C57BL/6 mice (*n* = 6) injected subcutaneously MB49 cells administrated with PBS or Asn. (**H**) Tumor growth curves and tumor weight of immunocompetent C3H mice (*n* = 6) injected subcutaneously with MBT2 cells administrated with PBS or Asn. Data were mean ± SD. Statistical significance was calculated by 2-tailed unpaired Student’s *t* tests for **E**–**H**; 1-way ANOVA for **A**–**D**. **P < 0.01, ***P < 0.001.

To investigate the effect of ASNS on tumor growth in vivo, ASNS-deficient mouse bladder cancer cells were injected subcutaneously into the flanks of immunocompromised mice. Our results demonstrated that loss of ASNS had no effect on tumor growth in BALB/c nude mice (Figure 1, A and B and Supplemental Figure 1, H and I). To determine the involvement of the immune system, we inoculated ASNS-deficient and control bladder cancer cells into syngeneic mouse hosts. Of note, silencing of ASNS inhibited tumor growth in both immunocompetent mice (Figure 1, C and D). To assess the effect of asparagine on the tumor growth in vivo, we subcutaneously injected murine bladder cancer cells into mice and followed with oral administration of PBS or asparagine. We observed no significant difference in immunocompromised mice administrated with asparagine (Figure 1, E and F). However, asparagine treatment promoted tumor growth in both immunocompetent murine bladder cancer models (Figure 1, G and H). Collectively, these data suggest that asparagine restriction could suppress tumor growth, which requires the presence of an intact immune system.

### Knockdown of ASNS potentiates recruitment and activation of CD8^+^ T cells.

**Figure 2:**
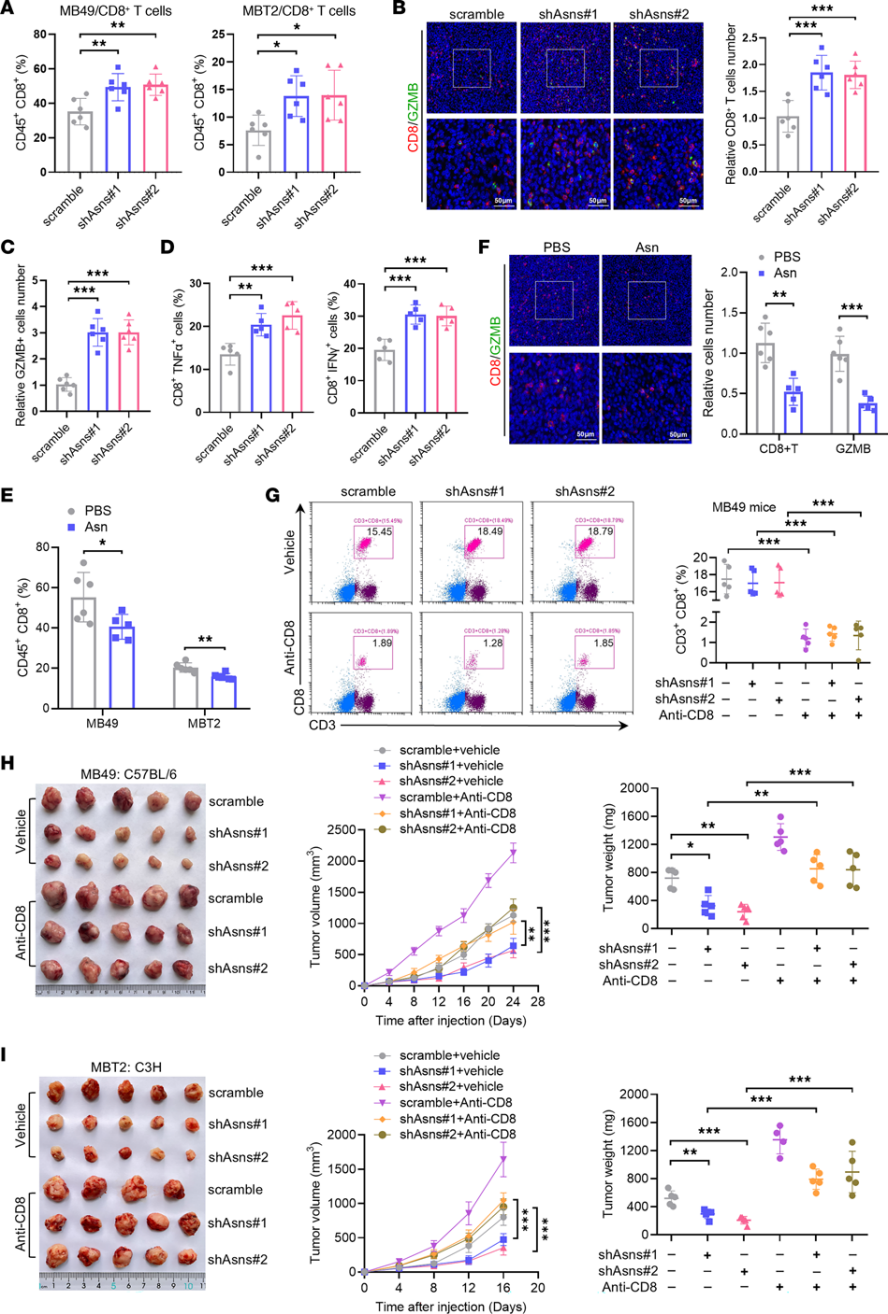
Knockdown of ASNS potentiates antitumor function of CD8^+^ T cells. (**A**) Tumor infiltrating CD8^+^ T cells from transplanted shAsns-MB49 tumors (*n* = 6) in C57BL/6 mice and shAsns-MBT2 tumors (*n* = 6) in C3H mice were analyzed by flow cytometry. (**B** and **C**) Representative images and quantification of immunofluorescence for CD8 (**B**) and GZMB (**C**) in scramble and shAsns-MB49 tumors (*n* = 6). Scale bars: 50 μm. (**D**) Flow staining and frequency of CD8^+^TNFα^+^ and CD8^+^IFNγ^+^ cells in shAsns-MB49 and control tumors (*n* = 5). (**E**) Tumor-infiltrating CD8^+^ T cells were analyzed by flow cytometry from transplanted MB49 and MBT2 tumors (*n* = 6) in syngeneic mice administrated with PBS or Asn. (**F**) Representative images and quantification of immunofluorescence for CD8 and GZMB in MB49 tumors administrated with PBS or Asn (*n* = 6). Scale bars: 50 μm. (**G**) C57BL/6 mice were subcutaneously inoculated with MB49 tumor cells and treated with anti-CD8 antibody. Flow cytometry analysis of CD8^+^ T cell content in peripheral blood of mice (*n* = 5) at the end of experiment. (**H**) Tumor growth curves and tumor weight from scramble and shAsns-MB49 tumor cells in C57BL/6 mice (*n* = 5) followed by intraperitoneal injection with anti-CD8 antibody. (**I**) Tumor growth curves and tumor weight from scramble and shAsns-MBT2 tumor cells in C3H mice (*n* = 5) followed by intraperitoneal injection with anti-CD8 antibody. Data were mean ± SD. Statistical significance was calculated by 2 tailed unpaired Student’s *t* tests for **E** and **F**. 1-way ANOVA for **A**–**D**; 2-way ANOVA for **G**–**I**. *P < 0.05, **P < 0.01, ***P < 0.001.

We next attempted to quantify immune effector cells in control and ASNS-deficient bladder tumors by flow cytometry. The gating strategy to analyze the population of immune cells in mouse-transplanted tumors is shown in Supplemental Figure 2A. We found that knockdown of ASNS could significantly increase CD8^+^ T cell infiltration in both tumors established by MB49 and MBT2 cells (Figure 2A). However, there were no consistent differences in CD4^+^ T cells and NK cells in both models (Supplemental Figure 3, A–D). Previous studies have shown that T cell exclusion from the tumor parenchyma is one of the mechanisms underlying immunosuppression in the TME and is associated with poor response to current immunotherapies (30, 31). Notably, depletion of ASNS also increased the infiltration of CD8^+^ T cells in tumor parenchyma (Figure 2B) and enhanced their cytokine production including GZMB, TNF-α, and IFN-γ (Figure 2, C and D, and Supplemental Figure 2B). On the contrary, asparagine treatment decreased the infiltration of CD8^+^ T cells and impaired function of CD8^+^ T cells to secrete GZMB (Figure 2, E and F and Supplemental Figure 3E).

To determine the extent to which ASNS inhibition promoted an antitumor reaction dependent on CD8^+^ T cells in vivo, CD8^+^ T cells were deleted using anti-CD8 antibody (Figure 2G and Supplemental Figure 3, F and G). We found that ASNS deficiency–mediated antitumor function was largely abolished in the CD8^+^ T cell–depleted group (Figure 2, H and I). Collectively, these results indicate that the tumor-suppressive effect of asparagine restriction might be mediated by tumor-infiltrating CD8^+^ T cells.

### Silencing of ASNS triggers RIG-I–induced IFN-I signaling.

**Figure 3:**
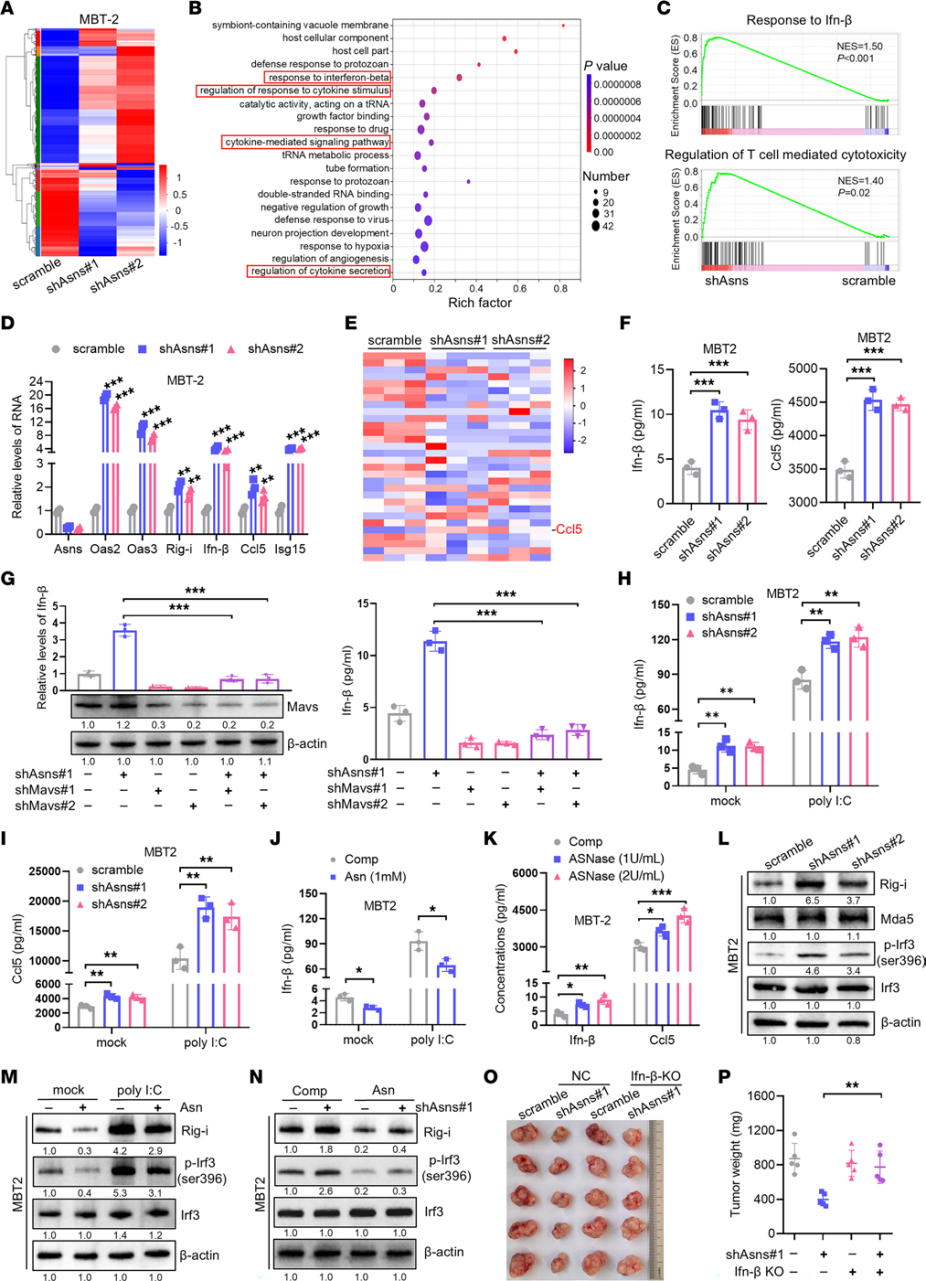
Silencing of ASNS activates RIG-I–induced IFN-I signaling. (**A**) Heat map depicted the differentially expressed mRNA in the indicated MBT2 cells. (**B**) Enrichment analysis for representative GO pathways in shAsns-mediated target genes. (**C**) GSEA plots of individual pathways enriched in shAsns-deficient MBT2 cells. (**D**) qRT-PCR showed the relative expression levels of ISGs genes in the indicated MBT2 cells. (**E**) Heatmap of multiple cytokines and chemokines detected by Luminex protein biochip testing system between Asns knockdown and the control groups in MBT2 cell culture supernatants. (**F**) ELISA experiment revealed the expression levels of Ifn-β and Ccl5 in culture supernatants of the indicated MBT2 cells. (**G**) qRT-PCR (left) and ELISA (right) assays showed the expression levels of Ifn-β in the indicated MBT2 cells. Western blot analysis of cell lysates from the indicated MBT2 cells. (**H** and **I**) Scramble or shAsns MBT2 cells were transfected with poly (I:C) (2 μg/mL) for 8 hours and the protein levels of Ifn-β (**H**) and Ccl5 (**I**) were determined by ELISA. (**J**) ELISA assay showed the expression levels of Ifn-β protein in the indicated MBT2 cells. (**K**) ELISA assay showed the expression levels of Ifn-β and Ccl5 in MBT2 cells treated with ASNase for 48 hours. (**L**) Western blot analysis of cell lysates from the MBT2 cells stably transfected with scramble and shAsns. (**M**) Western blot analysis of cell lysates from the indicated MBT2 cells. (**N**) Western blot analysis of cell lysates from the indicated MBT2 cells. (**O** and **P**) Tumor image and tumor weight of immunocompetent C57BL/6 mice (*n* = 5) injected subcutaneously with indicated MB49 cells. Data were mean ± SD. Statistical significance was calculated by 2 tailed unpaired Student’s *t* tests for **J**; 1-way ANOVA for **D**, **F**, **H**, **I**, and **K**; 2-way ANOVA for **G** and **P**. *P < 0.05, **P < 0.01, ***P < 0.001.

To further decipher the mechanism of immunoactivation mediated by ASNS inhibition, we performed RNA-seq analysis in ASNS-deficient MBT2 cells (Figure 3A and Supplemental Figure 4A). Gene ontology (GO) analysis and gene set enrichment analysis (GSEA) showed that differentially expressed genes were enriched in biological pathways related with the IFN pathway and adaptive immune response process, including response to IFN-I, regulation of T cell–mediated cytotoxicity, and so on (Figure 3, B and C and Supplemental Figure 4B). Then, we confirmed RNA-seq data through qRT-PCR that knockdown of ASNS elevated the expression of IFN-stimulated genes (ISGs) (e.g., OAS2, OAS3, RIG-I, CCL5, and ISG15) and IFN-β (Figure 3D and Supplemental Figure 4C). We also applied Luminex system multiple immunoassays to detect multiple cytokines and chemokines in cell culture supernatants from ASNS-deficient and control MBT2 cells. Supporting the results obtained in RNA-seq, knockdown of ASNS upregulated the expression of CCL5 and promoted its secretion (Figure 3E), which is robustly correlated with CD8^+^ T cell infiltration in solid tumors (32). Moreover, the secretion levels of IFN-β and CCL5 protein were improved in ASNS-deficient bladder cancer cells by using ELISA (Figure 3F and Supplemental Figure 4D). In contrast, asparagine attenuated the expression of IFN-β and CCL5 in bladder cancer cells (Supplemental Figure 4, E and F).

Previous studies have reported that several innate sensing pathways can stimulate the induction of IFN-I. For example, cGAS-STING of dsDNA sensors, RIG-I/MDA5-MAVS of dsRNA sensors, and Toll-like receptors (TLR3 and TLR4) of LPS sensors converge at the activation of TANK-binding kinase 1 (TBK1) and IFN regulatory factor 3 (IRF3), trigging the expression of IFN-I (33). However, IFN-I mediated by intracellular nucleic acid sensors are a key component to activate the immune system against cancer (34). Therefore, we inhibited the expression of pivotal nucleic acid sensors by shRNA-mediated silencing prior to knocking down ASNS to identify which sensor was essential for ASNS inhibition induced IFN-I activation. Intriguingly, abrogation of MAVS (Figure 3G), but not STING (Supplemental Figure 4G), substantially diminished the induction of IFN-β in response to ASNS inhibition. Therefore, we speculated that RIG-I/MDA5-MAVS pathway was essential for IFN-I activation upon ASNS inhibition. Then, we confirmed that knockdown of ASNS was sufficient to enhance IFN-β and CCL5 production following transfection of dsRNA mimic analogue poly (I:C) (Figure 3, H and I, and Supplemental Figure 4H), a synthetic dsRNA analogue (35). Conversely, exogenous asparagine decreased the expression of IFN-β and CCL5 induced by poly(I:C) (Figure 3J and Supplemental Figure 4, I and J). A previous study has reported that ASNase is a core component of the chemotherapy regimen for childhood ALL, which could participate in the catabolism of asparagine (36). Notably, the expression levels of IFN-β and CCL5 were higher in bladder cancer cells treated with ASNase (Figure 3K and Supplemental Figure 4K).

Accumulating studies have revealed that endogenous dsRNA is responsible for activating the cytoplasmic dsRNA sensors and triggering an IFN-I response in several types of cancer cells (37, 38). We investigated the level of endogenous dsRNAs using a dsRNA-specific antibody and found that the depletion of ASNS had no obvious effect on the level of endogenous dsRNA (Supplemental Figure 5A). Of note, knockdown of ASNS or asparagine could alter the expression of IFN-β and CCL5 upon poly(I:C) treatment. We speculated that ASNS loss or asparagine exerted their function by mediating dsRNA sensors, but not dsRNA production. We further explored whether ASNS inhibition activated RIG-I/MDA5 signaling and found that knockdown of ASNS upregulated RIG-I expression and promoted the phosphorylation of IRF3 proteins, a transcription factor that induces IFN expression (Figure 3L and Supplemental Figure 5B). We also observed a reduction of the RIG-I expression under asparagine addition (Supplemental Figure 5C) but not aspartate addition (Supplemental Figure 5D). In addition, we demonstrated that poly (I:C) intensely increased the expression of RIG-I and p-IRF3 protein, which could be reduced by asparagine in bladder cancer cells (Figure 3M and Supplemental Figure 5E). Importantly, the upregulation of RIG-I and p-IRF3 protein levels induced by ASNS knockdown was considerably abolished by asparagine (Figure 3N and Supplemental Figure 5F). Collectively, these findings reveal that ASNS inhibition could activate RIG-I–induced IFN-β signaling in bladder cancer.

To further investigate whether IFN-β signaling is essential for ASNS inhibition–elicited antitumor immunity, we genetically abrogated IFN-β production in bladder cancer cells (Supplemental Figure 6A), which completely reversed ASNS deficiency–mediated inhibition of tumor growth in vivo (Figure 3, O and P and Supplemental Figure 6, B and C). In line with this, we blocked IFN-β signaling using anti-IFNAR1 antibody in vivo and also found that treatment with anti-IFNAR1 antibody markedly restored the growth retardation of ASNS-deficient tumors in C57BL/6 mice (Supplemental Figure 6, D–G). Taken together, these results strongly indicate that IFN-β signaling plays a pivotal role in ASNS deficiency–mediated antitumor immune responses.

### Cellular asparagine abundance affects RIG-I protein stability.

**Figure 4:**
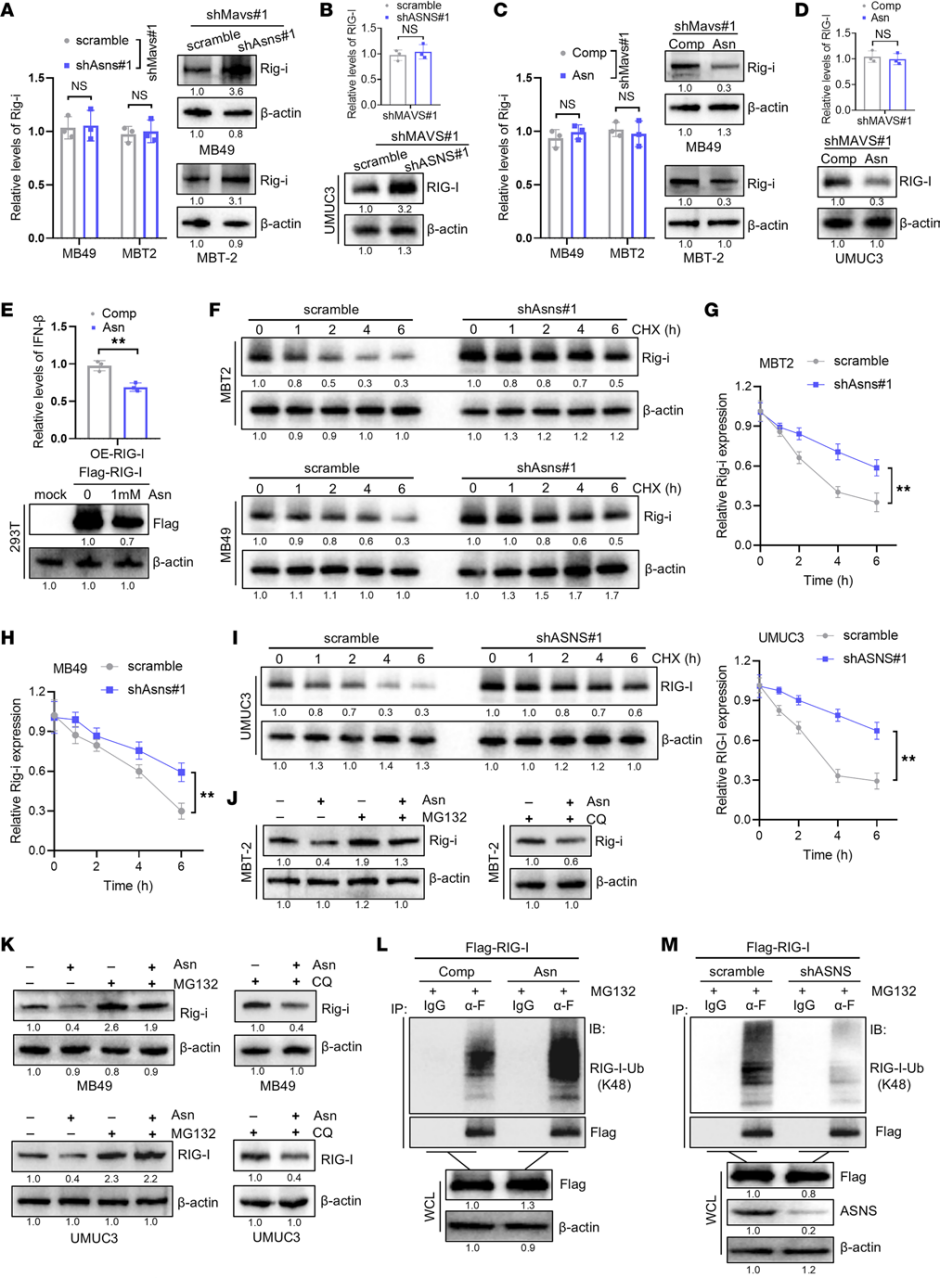
Asparagine facilitates the ubiquitination degradation of RIG-I. (**A**) qRT-PCR and Western blot showed the expression levels of Rig-i mRNA and protein in the indicated murine bladder cancer cells. (**B**) qRT-PCR and Western blot showed the expression levels of RIG-I mRNA and protein in the indicated UMUC3 cells. (**C**) qRT-PCR and Western blot showed the expression levels of Rig-i mRNA and protein in the indicated murine bladder cancer cells. (**D**) qRT-PCR and Western blot showed the expression levels of RIG-I mRNA and protein in the indicated UMUC3 cells. (**E**) qRT-PCR showed the expression levels of IFN-β in RIG-I–overexpressed HEK293T cells cultured in complete medium (Comp) and medium added Asn (1 mM) for 48 hours. Western blot showed the levels of RIG-I protein in the indicated groups. (**F**–**H**) Western blot revealed the degradation kinetics of Rig-i protein in the indicated Murine bladder cancer cells. The degradation rate of Rig-i protein was quantified by band intensity. (**I**) Western blot revealed the degradation kinetics of RIG-I protein in the indicated UMUC3 cells. The degradation rate of RIG-I protein was quantified by band intensity. (**J**) Western blot analysis showed the Rig-i expression in the indicated MBT2 cells. (**K**) Western blot analysis showed the RIG-I expression in the indicated MB49 or UMUC3 cells. (**L**) HEK293T cells were transfected with Flag-RIG-I and cultured in complete medium (Comp) or medium added Asn (1 mM) for 48 hours, followed by coimmunoprecipitation and immunoblotting analysis with the indicated antibodies. (**M**) HEK293T cells were transfected with Flag–RIG-I and with scramble or shASNS#1 for 48 hours, followed by coimmunoprecipitation and immunoblotting analysis with the indicated antibodies. Data were mean ± SD. Statistical significance was calculated by 2 tailed unpaired Student’s *t* tests for **A**–**E** and **G**–**I**. **P < 0.01.

We next sought to investigate the mechanism involved in ASNS inhibition–induced IFN-I pathway activation. The aforementioned results have depicted that ASNS inhibition could elevate the expression levels of mRNA and protein of RIG-I. Of note, previous studies have reported that RIG-I, as an ISG, could be strongly induced by IFN in a positive feedback manner (39, 40). Therefore, we inhibited the expression of MAVS by shRNA before knocking down ASNS to block the feedback effect of the IFN-I signaling on RIG-I and found that knockdown of ASNS upregulated the expression of RIG-I protein but not mRNA (Figure 4, A and B). Consistently, asparagine only decreased the level of RIG-I protein (Figure 4, C and D). Then, we transfected Flag–RIG-I plasmids in HEK293T cells and found that asparagine treatment attenuated the RIG-I and IFN-β expression (Figure 4E). Therefore, we hypothesized that RIG-I upregulation induced by ASNS inhibition was achieved by monitoring its protein layer.

Subsequently, we conducted we conducted the cycloheximide (a protein synthesis inhibitor) experiment and found that silencing of ASNS significantly extended the half-life of RIG-I protein, indicating that ASNS knockdown inhibited the degradation of RIG-I (Figure 4, F–I). In addition, the reduction of RIG-I protein induced by asparagine was abolished by proteasome inhibitor MG132 in bladder cancer cells, but not lysosome inhibitor CQ (Figure 4, J and K). Coimmunoprecipitation experiments indicated that asparagine markedly increased K48-linked ubiquitination of RIG-I (Figure 4L), whereas ASNS deficiency decreased K48-linked ubiquitination of RIG-I (Figure 4M). Collectively, these data suggest that knockdown of ASNS upregulates RIG-I expression by protecting it from degradation.

### Asparagine binds to RIG-I and enhances its ubiquitination by CBL.

**Figure 5:**
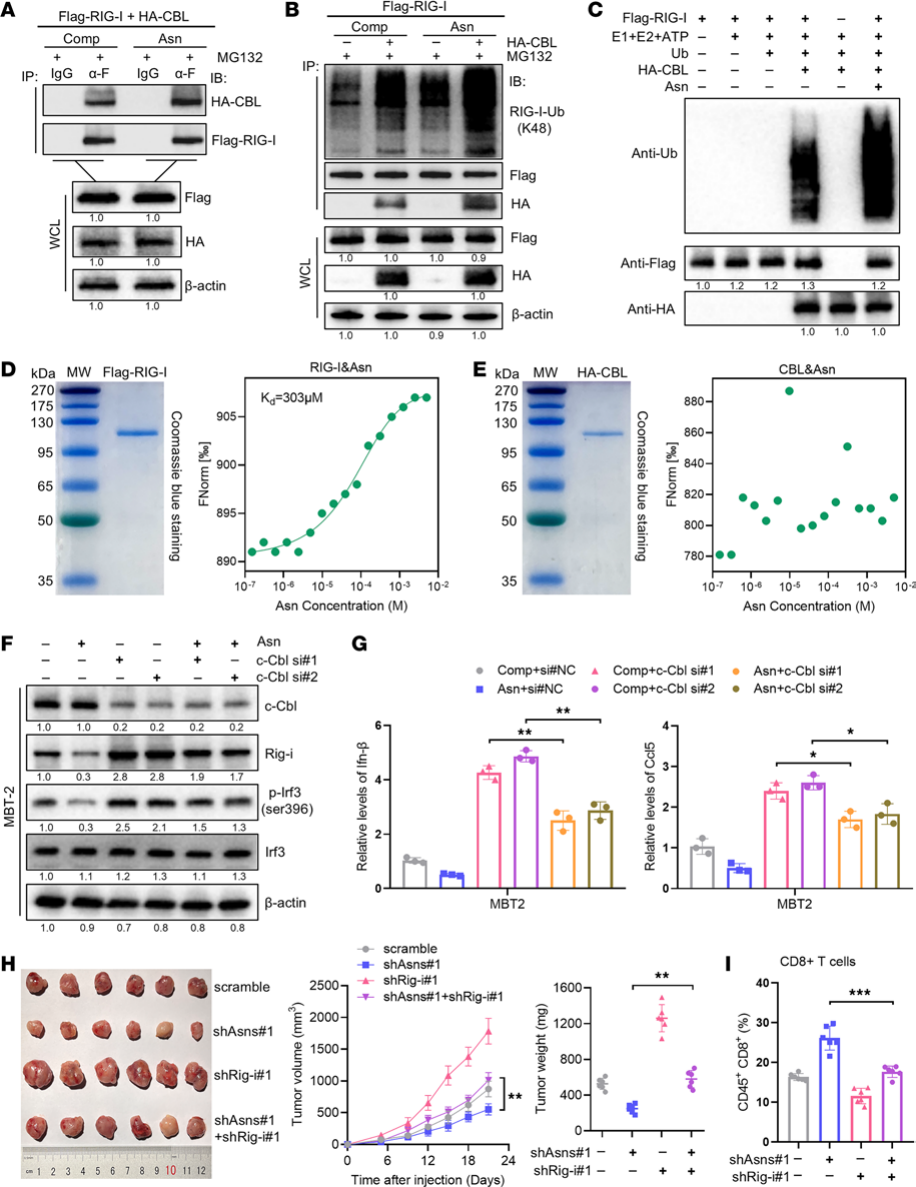
Asparagine promotes the CBL-mediated proteasomal degradation of RIG-I. (**A**) HEK293T cells were transfected with Flag-RIG-I and HA-CBL and cultured in complete medium (Comp) and medium added Asn (1 mM) for 48 hours, followed by coimmunoprecipitation and immunoblotting analysis with the indicated antibodies. (**B**) Flag-RIG-I–overexpressed HEK293T cells were cultured in complete medium (Comp) or medium added Asn (1 mM), and those cotransfected with a control and HA-CBL overexpression plasmid for 48 hours, followed by coimmunoprecipitation and immunoblotting analysis with the indicated antibodies. (**C**) Purified Flag-RIG-I proteins were incubated with the indicated proteins in the presence or absence of 1 mM Asn for 2 hours. Mixtures were analyzed by Western blot. (**D**) MST measurement of the interaction between Asn and purified RIG-I. Kd value was automatically by the curve fitting. (**E**) MST measurement of the interaction between Asn and purified CBL. (**F**) Western blot of the indicated proteins in MBT2 cells cultured in complete medium (Comp) or medium added Asn (1 mM), and those cotransfected with si-NC, si-Cbl#1 or si-Cbl#2. (**G**) qRT-PCR revealed the expression levels of Ifn-β and Ccl5 in MBT2 cells cultured in complete medium (Comp) or medium added Asn (1 mM), and those cotransfected with si-NC, si-Cbl#1, or si-Cbl#2. (**H**) Tumor growth curves and tumor weight of immunocompetent C3H mice (*n* = 6) injected subcutaneously with indicated MBT2 cells. (**I**) Tumor infiltrating CD8^+^ T cells from transplanted MBT2 tumors (*n* = 6) in C3H mice were analyzed by flow cytometry. Data were mean ± SD. Statistical significance was calculated by 2-way ANOVA for **G**–**I**. *P < 0.05, **P < 0.01, ***P < 0.001.

Increasing evidence indicates that the E3 ubiquitin ligases RNF122, RNF125, STUB1, and CBL play pivotal role in the RIG-I degradation via K48-linked ubiquitination (19, 41). We further determined how asparagine affects K48-linked polyubiquitination and suppression of RIG-I. Coimmunoprecipitation experiments indicated that asparagine promoted the association of CBL with RIG-I but not RNF122, RNF125, and STUB1 in mammalian overexpression system (Figure 5A and Supplemental Figure 7, A–C). Importantly, we demonstrated that asparagine could promote the polyubiquitination of RIG-I protein in CBL-overexpressed cells (Figure 5B). In contrast, CBL knockdown ablated the promotion of RIG-I polyubiquitination elicited by asparagine (Supplemental Figure 7D). Even though asparagine may trigger certain metabolic processes that can influence CBL-mediated RIG-I ubiquitination, it is worthwhile to detect whether asparagine acts directly on RIG-I. We thus tested the effect of asparagine on CBL-mediated ubiquitination of RIG-I in vitro. In the presence of E1, E2, ATP, and Ub, CBL promoted RIG-I ubiquitination, and asparagine addition strikingly enhanced ubiquitination (Figure 5C). Notably, microscale thermophoresis (MST) assay, an in vitro assay for direct interaction between proteins and small molecules, revealed that asparagine had a high affinity to bind to RIG-I with a Kd of 303 μM (Figure 5D), but did not show any affinity to CBL (Figure 5E). Thus, these results indicated that asparagine could directly bind to RIG-I and promote its degradation. We further observed that asparagine-mediated reduction of the RIG-I and p-IRF3 (Figure 5F and Supplemental Figure 7, E and F) as well as downregulation of IFN-β and CCL5 (Figure 5G and Supplemental Figure 7, G and H) were partially rescued by CBL knockdown.

Then, we detected the function of CBL knockdown on tumor growth and CD8^+^ T cell infiltration. The results revealed that silencing of CBL partially rescued the promotion of tumor growth and reduction of CD8^+^ T cell infiltration caused by asparagine (Supplemental Figure 7, I-L). Furthermore, we found that loss of RIG-I significantly increased the size of tumors derived from bladder cancer cells depleted of ASNS (Figure 5H). ASNS deficiency resulted in an obvious increase in infiltration of CD8^+^ T cells, which was attenuated by RIG-I knockdown (Figure 5I). Taken together, these data indicate that asparagine promotes the K48-linked ubiquitination of RIG-I by CBL, thereby attenuating antitumor immune response.

### Asparagine restriction sensitizes tumors to PD-1 blockade.

Tumor mutation burden has been proposed as a predictive biomarker for response to ICIs (42). Based on the high mutation profiles of bladder cancer, we therefore explored the therapeutic effect of PD-1 blockade monotherapy in vivo. We found that PD-1 blockade alone had minimal effect on tumor growth delaying effect (Supplemental Figure 8, A–D), consistent with previous report (43). Thus, it is necessary to improve the efficacy of ICIs by developing combination therapies.

**Figure 6:**
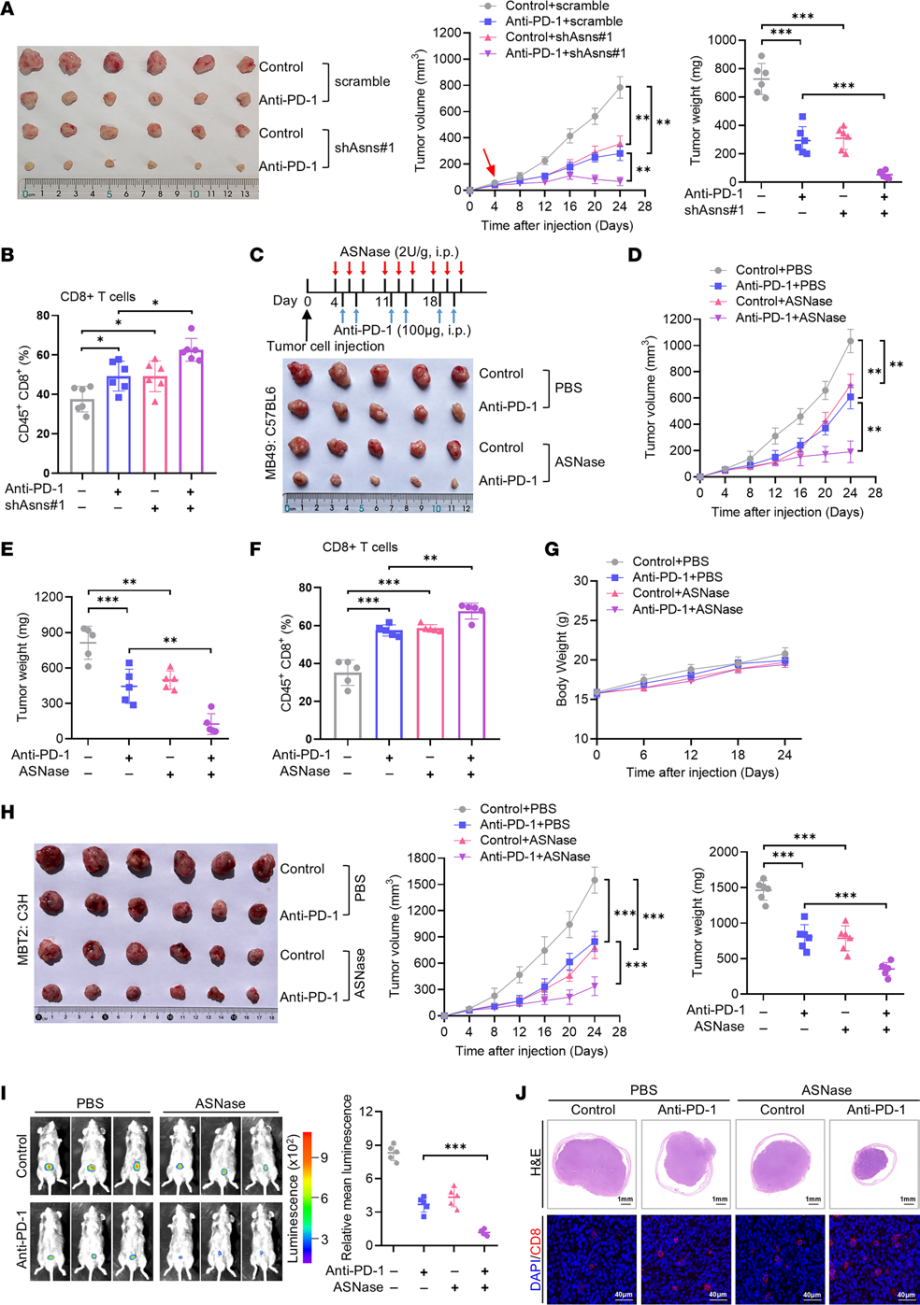
Asparagine restriction overcomes tumor resistance to PD-1 blockade in mouse model. (**A**) Tumor growth and tumor weight in immunocompetent C57BL/6 mice injected subcutaneously with MB49 cells stably transfected with scramble and shAsns#1 and treated with anti-PD-1 or isotype control (*n* = 6). (**B**) Flow cytometry showed the tumor infiltrating CD8^+^ T cells in MB49 tumors of indicated groups. (**C**–**E**) Schematic of ASNase therapy. Tumor growth and tumor weight in immunocompetent C57BL/6 mice injected subcutaneously with MB49 cells administrated with ASNase, and treated with anti-PD-1 or isotype control (*n* = 5). (**F**) Tumor infiltrating CD8^+^ T cells in MB49 tumors of indicated groups were analyzed by flow cytometry. (**G**) The body weights among different groups during experimental procedure. (**H**) Tumor image and tumor weight in immunocompetent C3H mice injected subcutaneously with MBT2 cells administrated with ASNase, and treated with anti-PD-1 or isotype control (*n* = 6). (**I**) Representative luminescence images and histogram analysis of bioluminescence intensity in C57BL/6 mice injected orthotopically with luc-labeled MB49 cells with the treatment of ASNase and anti-PD-1 antibody. (**J**) Representative H&E staining and immunofluorescence for CD8 of tumors in the indicated groups. Scale bars (H&E): 1 mm. Scale bars (IF): 40 μm. Data were mean ± SD. Statistical significance was calculated by 2-way ANOVA for **A**, **B**, **D**, **E**, **F**, **H**, and **I**. *P < 0.05, **P < 0.01, ***P < 0.001.

Considering that ASNS inhibition could enhance CD8^+^ T cell infiltration and promote its activation, we further explored whether ASNS could affect the efficacy of anti–PD-1 treatment preclinically. We conducted tumor growth experiments with anti–PD-1 antibody in syngeneic mice inoculated with ASNS-deficient MB49 tumor cells. Our results demonstrated that anti–PD-1 antibody synergized with ASNS deficiency in suppressing tumor growth (Figure 6A). Flow cytometry analysis also showed that ASNS knockdown combination with anti–PD-1 treatment significantly increased the infiltration of CD8^+^ T cells (Figure 6B). More importantly, ASNase markedly enhanced the antitumor effects of PD-1 blockade in the subcutaneous tumor models established by MB49 cells (Figure 6, C–E). Further analysis of immune infiltration revealed that combination treatment could also promote CD8^+^ T cell infiltration (Figure 6F). In addition, there was no obvious difference in body weight among the 4 groups of mice (Figure 6G). Consistent with the observations made in MB49 tumor mice, a notable delay in tumor growth was observed in MBT2 tumor mice treated with the ASNase and PD-1 blockade (Figure 6H and Supplemental Figure 8, E and F). Furthermore, since the subcutaneous model does not faithfully recapitulate the microenvironment of bladder cancer, we applied the orthotopic bladder tumor model along with ASNase and anti–PD-1 treatment. As shown in Figure 6, I and J, ASNase alone reduced tumor growth, and the combination of anti–PD-1 and ASNase more efficiently suppressed tumor progression. Immunofluorescence staining results indicated that anti–PD-1 antibody increased CD8^+^ T cell number infiltration in tumors, which could be strengthened upon ASNase treatment (Figure 6J). Altogether, these results indicate a potent synergy between ASNS inhibition and PD-1 blockade in controlling tumor growth in bladder cancer.

### Upregulated ASNS leads to immunotherapy resistance in bladder cancer.

**Figure 7:**
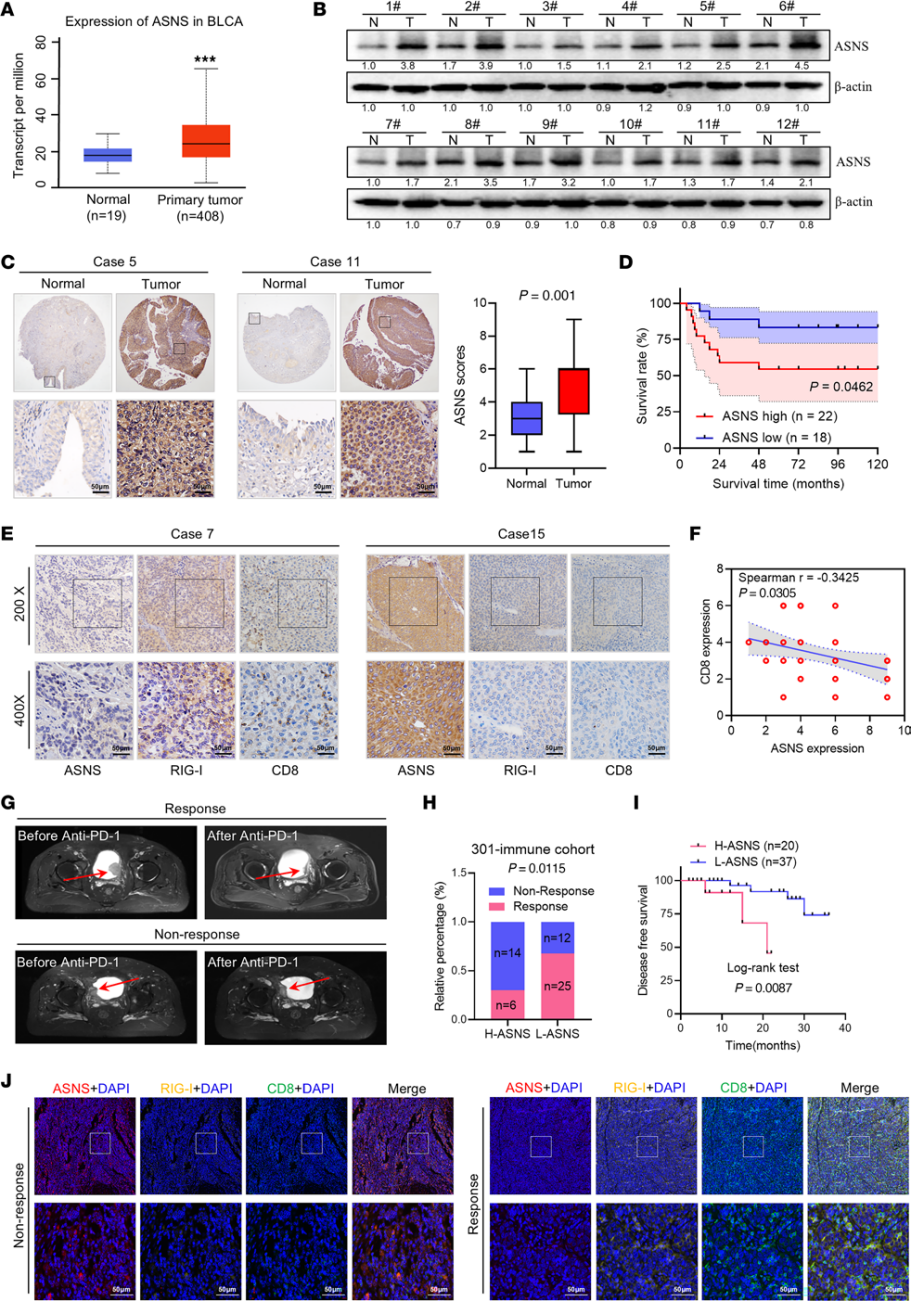
Upregulated S leads bladder cancer resistance to ICIs. (**A**) mRNA levels of ASNS in bladder cancer samples from TCGA cohort. (**B**) Western blot showed the expression levels of ASNS in our paired samples of bladder cancer. (**C**) Representative IHC staining and quantification revealed ASNS expression in paired samples of bladder cancer from our cohort (*n* = 40). Scale bar: 50 μm. (**D**) Kaplan-Meier analysis of the overall survival of 40 patients with high or low expression of ASNS. (**E**) Representative IHC staining of ASNS, RIG-I, and CD8 in bladder cancer samples from our cohort. (**F**) Correlation analysis of ASNS expression and CD8 expression in bladder cancer clinical samples (*n* = 40). (**G**) Representative MRI image for patients with response and nonresponse after ICI treatment in our hospital (301-immune cohort, *n* = 57). (**H**) The relationship between ASNS expression level and immunotherapy efficacy in the 301-immune cohort. (**I**) Disease-free survival of patients with different ASNS IHC scores in our immune cohort. (**J**) Representative multicolor IF images for ASNS (red), RIG-I (yellow), CD8 (green), and DAPI (blue) in patients in the response and nonresponse groups. Scale bars: 50 μm. Data were mean ± SD. Statistical significance was calculated by 2-tailed unpaired Student’s *t* tests for **A**; Paired Student’s t-tests for **C**; Chi-square test for **H**; Survival analysis of **D** and **I** was performed by the log-rank test. ***P < 0.001.

To further explore the profiles of ASNS in bladder cancer, we mined the TCGA dataset and found that the mRNA level of ASNS was significantly upregulated in bladder cancer (Figure 7A). We then confirmed that the level of ASNS protein was higher in bladder cancer tissues than that in paired normal bladder tissues (Figure 7B). Moreover, IHC staining in bladder cancer tissue array show that the expression of ASNS was upregulated in tumor tissues (Figure 7C). Further Kaplan-Meier analysis revealed that high expression of ASNS was associated with poor overall survival for patients with bladder cancer (Figure 7D). Next, we verified the correlation between tumor ASNS, RIG-I, and CD8 expression in tissues of patients with cancer. IHC staining exhibited a negative correlation between tumor CD8 expression and ASNS expression in human bladder cancer clinical samples (Figure 7, E and F). Similar to CD8, we found that tumor RIG-I expression was also inversely correlated with ASNS protein expression (Supplemental Figure 9A). Furthermore, the infiltration degree of CD8^+^ T cells showed a positive correlation with RIG-I (Supplemental Figure 9B).

To further assess the clinical value of ASNS in ICI therapy, we analyzed an immunotherapy cohort containing 57 patients with bladder cancer treated with ICIs from our hospital (301-immune cohort). The expression of ASNS at baseline was lower in responders to ICIs compared with nonresponders (Figure 7, G and H and Supplemental Figure 9C). Meanwhile, low levels of ASNS expression were associated with improved progression-free survival in these patients (Figure 7I). Moreover, multicolor IF images confirmed a negative correlation between ASNS expression and RIG-I expression and CD8^+^ T cell infiltration (Figure 7J), revealing the mechanism of response or nonresponse to ICI treatment. Thus, these data indicated that ASNS could be a predictive biomarker for bladder cancer ICI therapy.

## Discussion

Recently, ICI therapy has become a promising therapeutic strategy with bladder cancer due to its high levels of mutational burden (2). However, only a minority of patients benefit from this strategy, while a considerable proportion of patients either fail to respond or progress rapidly to resistance after initial response (8). Therefore, finding predictive biomarkers for increasing immune response and developing therapeutic strategies to potentiate immunotherapy efficacy are urgently needed. Our study found that ASNS expression is a predictive biomarker for immunotherapy response in bladder cancer, and FDA-approved ASNase dramatically boosts the antitumor efficacy of anti–PD-1 therapy. Anti–PD-1 therapy combined with ASNase in advanced bladder cancer is worthy of investigation in future clinical trials. Previous studies showed that asparagine availability favors tumor cell proliferation, metastasis, and survival ability in various types of cancers (27–29), while we found that knockdown of ASNS or asparagine addition had no obvious effect on proliferation or migration of bladder cancer cells, indicating cancer type–specific functions in asparagine metabolism. However, further research should focus on the underlying mechanisms of ASNS upregulation in bladder cancer, which may help to advance understanding its special role.

Modulation of amino acid metabolism has emerged as a way to reprogram the tumor microenvironment and has shown great promise for cancer treatment, but, so far, major studies have focused on immune cells themselves (20–22, 44, 45). As a nonessential amino acid, asparagine uptake and biosynthesis enable CD8^+^ T cell activation (early phase) (24–26), while exerting opposing effects during differentiation (late phase) (26). These studies revealed asparagine as a critical metabolic node to shape T cell effector functions and antitumor responses. However, it is unclear whether asparagine has any effect on tumor-intrinsic functions to regulate immune responses. Our data demonstrate that limiting asparagine by knockdown of ASNS or treatment with ASNase in bladder cancer facilitates RIG-I–mediated IFN-I signaling, promoting the intratumoral CD8^+^ T cell infiltration and PD-1 blockade efficacy. Together, these encouraging results imply that remodeling of asparagine metabolism is critical for relieving tumor-mediated immunosuppression and is an effective way to enhance antitumor immunity.

Increasing evidences indicate that the exclusion of CD8^+^ T cells in TME is associated with poor response to immunotherapy (30, 31). Herein, we demonstrated that knockdown of ASNS could potentiate recruitment and activation of CD8^+^ T cells in bladder cancer. However, ASNS deficiency–mediated antitumor response was not completely reversed by CD8 depletion in syngeneic mice. In contrast, depletion of IFN-β and anti-IFNAR1 antibody treatment almost completely reversed tumor regression mediated by ASNS knockdown in syngeneic mice, suggesting that other types of immune cells besides CD8^+^ T cells might also contribute to ASNS loss–triggered antitumor immunity. Previous studies have shown that cytotoxic CD4^+^ T cells and NK cells could mediate the antitumor responses of bladder cancer (46, 47). We hypothesized that, although silencing of ASNS showed no obvious difference in the number of CD4^+^ T cells and NK cells in TME, it may alter their status and function.

Posttranslational modification of the RLRs directly regulates their expression and activity, subsequently modulating the downstream IFN-I signaling (48, 49). Of note, K48-linked ubiquitylation catalyzed by several E3 ligases, such as RNF122, RNF125, CBL, or STUB1, contributes to the degradation of RIG-I, thereby inﬂuencing pathway activation (41, 50–52). By far, little is known about whether nutrient metabolites are crucially involved in the regulation of RIG-I-IFN signaling. In this study, we identify asparagine as a metabolic regulator of RIG-1 stability and verify that asparagine directly binds to RIG-I, promoting CBL-mediated polyubiquitination and proteasomal degradation of RIG-I. Previous studies report the metabolism-independent role for asparagine in the direct modulation of LKB1 and LCK kinase activity (24, 29). We uncover that asparagine could be an endogenous metabolite binding to RIG-I and regulate its stability. However, further structural study may be required to comprehensively understand how asparagine acts on the RIG-I-CBL complex.

In conclusion, our study establishes a critical role of asparagine in limiting IFN-1 signaling and identifies RIG-I as a direct sensor of asparagine, which furnishes a rationale for future clinical applications of the combinatorial use of ASNase and ICIs in bladder cancer.

## Methods

### Sex as a biological variable.

Male and female human bladder cancer samples were analyzed. Male and female mice were used in all mouse studies. In this study, sex was not considered as a biological variable.

### Animals.

6- to 8-week-old C3H mice were purchased by Beijing Vital River Laboratory Animal Technology Co. Ltd. C57BL/6J mice and BALB/c nude mice aged 6–8 weeks were purchased from GemPharmatech Co. Ltd). All mice were maintained at room temperature with free access to food and water with a 12-h light/dark cycle in a barrier facility.

### Cell lines and clinical samples.

The bladder cancer cell lines UMUC3 were obtained from the ATCC. HEK293T cells were also purchased from the ATCC. The murine malignant bladder cell lines MB49 and MBT2 were purchased from Meisen CTCC. The UMUC3, MB49, and MBT2 cells were cultured in DMEM (Procell). All the media were supplemented with 10% FBS (Procell) and 1% penicillin and streptomycin (Procell). The condition of the incubator was set as 37°C with 5% CO_2_. All cell lines were routinely confirmed negative for *Mycoplasma* contamination. Resected human bladder cancer tissues and their corresponding clinical information were acquired from the Department of Urology of the Chinese PLA General Hospital. The histologic and pathologic type of all tissues were confirmed by 3 experienced pathologists independently. For the 301-immune cohort, a total of 57 patients with bladder cancer receiving ICIs were included from Chinese PLA General Hospital. ICI treatment efficacy was inspected by at least 3 pathologists based on the Response Evaluation Criteria in Solid Tumors (RECIST). The samples were obtained with written informed consent of all patients before the research started.

### Plasmids construction and transfection.

Short hairpin RNA sequences against mouse Asns, Rig-i, Mavs, c-Cbl and human ASNS were synthesized by Biomed and were cloned into the pLKO.1 vector. Knockdown plasmid against mouse Sting has been constructed in our previous study (53). The overexpression vector of RIG-I was kindly gifted from Weina Zhang (Academy of Military Medical Sciences). The human RNF122, RNF125, STUB1, and CBL cDNA were synthesized by Biomed, which were cloned into pCMV-HA vector to construct the overexpression plasmid. sgRNA sequence of Ifnb was synthesized by Biomed and was cloned into the lentiCRISPR v2 vector. The lentiviral vectors were transfected in HEK293T cells with the PAX2 and VSVG packaging plasmid. After a 48 or 72 hour transfection, supernatants were collected and concentrated with Lentivirus Concentration Reagent (GenStar) for overnight in 4°C. To construct the stably transfected cell lines, cells were infected with relative lentiviruses for 24 hours, then were screened with puromycin for 1 week. shRNA sequences are also listed in Supplemental Table 1.

### Cell viability assays.

For the cell proliferation assay, the bladder cancer cells were seeded in 96-well plates in 100 μL complete culture media for various time periods (2,000 cells per well). Cell Counting Kit-8 assay (Dojindo) was performed to measure cell viability according to the manufacturer’s instructions.

### Animal experiments.

For the subcutaneous tumorigenesis experiments, 6- to 8-week-old BALB/c nude mice, C57BL/6 and C3H mice were used. MB49 (5 × 10^5^ per mouse) and MBT2 (1 × 10^6^ per mouse) cells stably transfected with the pLKO.1-shAsns#1 plasmid or pLKO.1-Vector plasmid were subcutaneously injected into the right flank of each mouse. When a tumor was palpable, it was measured using a caliper every 3–4 days, and its volume was calculated according to the formula volume = length × width^2^ × 0.5. A total of 4 days after implantation, mice were treated with 100 μg of either control IgG2α (Bioxcell, BE0085) or anti-PD-1 antibody (Bioxcell, BE0146). The drugs were administered twice a week for 3 weeks. For Asparagine experiment, mice were fed with drinking water with or without 10 mM Asparagine (Solarbio). For drug combination experiments, 5-week-old C57BL/6 or C3H mice were inoculated subcutaneously with MB49 cells (5 × 10^5^ per mouse) or MBT2 cells (1 × 10^6^ per mouse). Next, mice received treatment i.p. with vehicle or ASNase (2 U/g) (ProSpec) 3 times a week for 3 weeks. In addition, 100 μg of anti–PD-1 antibody were injected beginning on day 5 twice a week for 3 weeks in combination with the ASNase. For neutralization of IFNAR1, tumor-bearing mice were injected i.p. with 200 μg/mouse anti-IFNAR1 (BioXcell, BE0241) at on days 0, 7, 14, and 21.

### Quantitative real-time PCR.

Briefly, total RNA was isolated from bladder cancer cells in each condition using FastPure Cell/Tissue Total RNA Isolation Kit V2 (Vazyme) according to the manufacturer’s instructions. RNA of each sample was complementarily reversed to cDNA by HiScript III RT SuperMix for qPCR (Vazyme). cDNA product of each sample was used as template to conduct quantitative PCR by ChamQ Universal SYBR qPCR Master Mix (Vazyme). Quantitative RT-PCR was performed using CFX96 Real-Time PCR System (Bio-Rad). The fold changes of gene expression were calculated after being normalized to 18s or ACTB. The primer pairs used in this study were listed in Supplemental Table 1.

### Western blotting analysis.

Briefly, cells were lysed by using RIPA buffer containing proteinase inhibitors cocktail and PMSF on ice for 30 minutes. Protein samples were quantified using BCA protein assay kit (Solarbio) and boiled in 5 × loading buffer for 10 minutes at 95°C. Protein lysates were resolved by SDS-PAGE and transferred onto polyvinyldifluoride (PVDF) membrane. Then, the membranes were blocked by 5% skimmed milk and probed with primary antibodies against Actin (Bioeasytech, BE0033), Asns (Proteintech, 14681-1-AP), Sting (Proteintech, 19851-1-AP), Mavs (Proteintech, 14341-1-AP), Rig-i (CST, 4200), Mda5 (ABclonal, A2419), Irf3 (CST, 4302), p-Irf3 (CST, 29047) and CBL (Proteintech, 25818-1-AP) at 4°C overnight. Membranes were washed with TBST 3 times and then incubated with HRP-conjugated anti-rabbit (Proteintech, SA00001-2) or anti-mouse (Proteintech, SA00001-1) secondary antibodies at room temperature for 1 hour. Finally, the protein bands were visualized using ECL substrate kit via QuickChemi 5200 Imaging System.

### Migration assays.

For migration assays, approximately 4 × 10^4^ cells in 200 μL serum-free culture medium were seeded in the upper chamber of 24-well Transwell chamber system. About 600 μL culture medium with 10% FBS was added to the lower chamber. After incubation for 24 hours, cells were fixed in paraformaldehyde for 15 minutes and then stained with 0.1% crystal violet for 30 minutes. The migrated cells were counted in 3 randomly selected fields.

### FACS analysis with tumor-infiltrating immune cells.

Mice harboring tumors were collected after inoculation, weighed, and mechanically minced and digested in DNase I and collagenase IV (Sigma-Aldrich) for 60 minutes at 37°C. The dissociated cells were filtered by 70 μm cell strainers (BIOFIL) to obtain single cell suspension for flow cytometry analysis. Tumor cells were washed with RPMI-1640 medium and lysed with RBC Lysis Solution (Beyotime). Then, BV510 anti-mouse CD45 (BioLegend, 103137), BV605 anti-mouse CD3 (BioLegend, 100237), Alexa Fluor 488 anti-mouse CD4 (BioLegend, 100423), APC/Fire 750 anti-mouse CD8a (BioLegend, 100766), and APC-mouse NK1.1 (BioLegend, 108710) were used to stain cell members for 30 minutes in the dark. Flow cytometry was used to detect stained cells, and FlowJo software was used to analyze the data.

For cytokine staining, tumor cells were digested into single-cell suspension and centrifuged for 5 minutes at 400*g*. All cells were then cultured in RPMI-1640 with 10% FBS and penicillin/streptomycin with GolgiPlug (BD, 554724) and eBioscience (Thermo Fisher Scientific, 00-4970-93) for 3 hours at 37°C. Cells were washed and live/dead staining (Thermo Fisher Scientific, 65-0865-14) and BV421 anti-mouse CD45 (BioLegend, 103133) and PerCP anti-mouse CD8 (BioLegend, 100731) staining were performed. Then cells were fixed for 1 hour at room temperature in Fixation/Permeabilization Concentrate and washed in permeabilization buffer (Thermo Fisher Scientific, 00-5523-00). APC anti-mouse TNFα (BioLegend, 506307) and PE anti-mouse IFNγ (BD, 554412) staining was performed in permeabilization buffer at room temperature for 30 minutes. Cells were washed and resuspended in FACS buffer for analysis on flow cytometer.

### IHC staining.

Paraffin-embedded tissues were sectioned and used to detect the expression of ASNS, RIG-I, and CD8. IHC staining was conducted as described previously. Briefly, samples were incubated with primary antibodies, including anti-ASNS (Proteintech, 14681-1-AP), anti-RIG-I (ABclonal, A21421), and anti-mCD8a (CST, 98941) at 4°C overnight. Sections were incubated with enzyme-conjugated secondary antibody followed by washing 3 times with PBS. After washing, sections were incubated with DAB substrate kit. Sections were counterstained with hematoxylin. The stained bladder cancer tissues were scored based on a scoring method as follows: staining intensity was scored 0 (negative), 1 (low), 2 (moderate), and 3 (high). Staining range was scored 0 (0% stained), 1 (1%–25% stained), 2 (26%–50% stained), and 3 (51%–100% stained). The total score was calculated according to the formula score = intensity score × percentage score.

### Immunofluorescence.

For dsRNA immunofluorescence, cells were plated on glass coverslips in a 24-well plate. After overnight cultivation, cells were washed 3 times with PBS and then fixed with 4% formaldehyde at room temperature for 20 minutes and permeabilized with 0.5% Triton-X100 in PBS for 5 minutes at room temperature. Then, cells were blocked by 1% BSA and incubated in the J2 antibody (SCICONS, 10010200) at 4°C overnight. The following day, cells were washed 3 times with PBST and incubated with ABflo 594-conjugated Goat anti-Mouse IgG (ABclonal, AS054) for 1 hour at room temperature, followed sealing with antifade mounting medium containing DAPI.

For multiplex immunofluorescence staining of tissues, tumors were captured and fixed in 10% formalin overnight and embedded in paraffin. The embedded tissues were sectioned into slices with a thickness of 4 μm, deparaffinized, hydrated, and antigen retrieved in 10 mmol/L sodium citrate for 20 minutes in a microwave oven, then cooled to room temperature slowly. Sections were permeabilized with 0.5% Triton X-100 in PBS for 5 minutes and then blocked with 2% BSA in PBS for 1 hour at room temperature. The sections were incubated with anti-mCD8a (CST, 98941), anti-GZMB (proteintech, 13588-1-AP), anti-hCD8a (CST, 70306), ASNS (proteintech, 14681-1-AP) and anti-RIG-I (ABclonal, A21421) overnight at 4°C. Subsequently, sections were washed with PBS and incubated with fluorescein-conjugated secondary antibody at room temperature for 1 hour. After incubation with DAPI, the slides were imaged and visualized using CLSM 600 confocal laser scanning microscope (Sunnysoptop).

### In vivo experiments with CD8 depletion.

MB49 (5 × 10^5^ per mouse) and MBT2 (1 × 10^6^ per mouse) cells stably transfected with the pLKO.1-shAsns#1 plasmid, pLKO.1-shAsns#2 plasmid, or pLKO.1-Vector plasmid were subcutaneously injected into 6- to 8-week-old C57BL/6 mice and C3H mice, respectively. To evaluate the role of CD8^+^ T cells in mice, the 200 μg of anti-CD8a antibody (MCE, HY-P99129) was injected intraperitoneally on days –3, 0, 3, 8, and 11. Equal amounts of IgG isotype antibodies were injected as a control. Tumor volume and growth curve were detected as mentioned above.

### RNA-seq and GSEA.

Total RNA isolated using FastPure Cell/Tissue Total RNA Isolation Kit V2 (Vazyme. RNA-seq analysis was performed by Majorbio Bio-pharm Technology Co. Ltd. Transcriptome library was prepared following TruSeq RNA sample preparation Kit from Illumina using 1 μg of total RNA. DEGs with |log2FC| greater than 1.5 and adjusted *P* value less than or equal to 0.05 were considered to be significantly different expressed genes. The data were analyzed on the online platform of Majorbio Cloud Platform. Sequencing results were deposited in the Gene Expression Omnibus database (GEO GSE270353). The GSEA algorithm was used to analyze the biological functions of the genes based on IFN-I–related molecular signatures collected from Molecular Signatures Database (MSigDB).

### Luminex liquid suspension biochip detection.

MBT2 cells stably transfected with the pLKO.1-shAsns#1 plasmid or pLKO.1-Vector plasmid were cultured in the 6-well plates. After 24 hours, cell culture supernatants were collected and the inflammatory cytokines and chemokines were detected (including: CCL1, CCL2, CCL3, CCL4, CCL5, CCL7, CCL11, CCL12, CCL17, CCL19, CCL20, CCL22, CCL24, CCL27, CXCL1, CXCL5, CXCL10, CXCL11, CXCL12, CXCL13, CXCL16, CX3CL1, IL-2, IL-4, IL-6, IL-10, IL-16, GM-CSF, IFN-γ, IL-1β, and TNF-α) by a Luminex protein biochip testing system (Wayen Biotechnologies) according to manufacturer’s instruction. The data were measured and collected by the Luminex 200 system. The results were showed in Supplemental Table 2.

### ELISA.

Cell culture supernatants were collected from 6-well plates, and the presence of the cytokine/chemokine proteins was determined using the Mouse IFN-β and Mouse CCL5 ELISA kit (Elabscience) according to the manufacturer’s instructions. The concentrations of different cytokine/chemokine proteins were calculated based on OD values at a detection wavelength of 450 nm.

### Co-IP.

HEK293T cells were transfected with Flag-RIG-I and HA-RNF122, HA-RNF125, HA-STUB1, and HA-CBL together with Asparagine (Solarbio) for 42 hours and cultured in medium containing MG132 (MCE) for a further 6 hours before coimmunoprecipitation. Then the cells were lysed with NP-40 buffer plus complete protease inhibitor PMSF and cocktail, and samples were cleaved on ice for 30 minutes. The lysates were centrifuged at 10,000*g* for 10 minutes at 4°C. About 5%–10% of the supernatants were used as input group, and the remaining samples were divided equally into IgG group and Flag group. The protein A/G beads were added in IgG or Flag antibodies at 4°C for 2 hours. Then the supernatants were incubated with antibody-beads mix at 4°C overnight. Proteins interacting with beads were removed by resuspending the beads in 1× SDS-PAGE loading buffer followed by heating at 95°C for 10 minutes. Proteins were separated by SDS-PAGE, followed by immunoblotting analysis with the Flag (proteintech, 66008-4-Ig), HA (proteintech, 51064-2-AP), and Ub-K48 (ABclonal, A3606) antibodies.

### In vitro ubiquitination assay.

Flag-RIG-I and HA-CBL were expressed in HEK293T cells and purified using Flag and HA beads, respectively. The reaction was carried out at 37 °C for 2 hours in 20 μL reaction buffer (40 mM Tris-HCl at pH 7.6, 2.5 mM Mg^2+^-ATP, 2 mM dithiothreitol) containing the following components: 50 μM of ubiquitin, 100 nM E1, 1 μM E2, 2 μM HA-CBL, and 5 μM Flag-RIG-I. The samples were stopped by adding SDS and boiled for 10 minutes, then resolved by SDS-PAGE followed by immunoblot analysis using a monoclonal anti-Ub antibody.

### MST assay.

MST is a biomolecular interaction analysis technology that measures the affinity between a ligand and a target molecule (54). Briefly, the HEK293T cell lysate was collected after 48 hours transfection with the Flag-RIG-I or HA-CBL plasmid. We purified Flag-tagged RIG-I and HA-tagged CBL by acid elution method. In total, 16 titration series of asparagine were prepared beginning at a concentration of 5 mM and mixed with purified protein. All the samples were loaded into MST NT.115 standard glass capillaries and measurement was carried out using the MO control software of Monolith NT.115.

### Orthotopic bladder tumor model.

An orthotopic bladder tumor mouse model was established as described previously (55) with minor modifications. In brief, under anesthesia, the C57BL/6 mice were placed in a supine position on a thermostatic blanket. Then, a 1-cm midline incision through the skin of the abdominopelvic region was made to locate the urinary bladder. Luc-labeling MB49 cells were resuspended with 50% Matrigel (Lablead), mixed with PBS (5 × 10^3^ cells per mouse), and were injected into the bladder wall of mice using insulin syringe. The bladder was placed back inside while ensuring that all other organs were in their proper position. Mice were left for at least 5 days for tumor development. After tumor volume was measured by living image, mice were randomly allocated into 4 groups for ASNase or anti–PD-1 treatment. For bioluminescent imaging, mice were given 200 μL (15 mg/mL) D-luciferin potassium salt (Lablead) intraperitoneally and imaged on a live imaging system (Berthold, LB983).

### Statistics.

The results are presented as the mean ± SD. Kaplan-Meier method and log-rank test were used to calculate overall survival rates. The significance of intergroup differences was determined with 2-tailed Student’s *t* test, 1-way ANOVA, or 2-way ANOVA. Statistical significance was assessed using GraphPad Prism software. A difference of *P* < 0.05 was considered statistically significant.

### Study approval.

All bladder cancer tissue samples used in this study were obtained from patients with their informed consent, and the use of these samples was approved by the IRB of the Chinese PLA General Hospital. All animal experiments were performed in accordance with institutional regulations and approval by the Institutional Animal Care and Use Committee of the Chinese PLA General Hospital.

### Data availability.

RNA-seq data reported in this paper were deposited in the Gene Expression Omnibus database (GEO GSE270353). Values for all data points in graphs are reported in the Supporting Data Values file. All unique/stable reagents generated in this study are available from the corresponding author with a completed material transfer agreement.

## Author contributions

WW was responsible for conceptualization, data curation, formal analysis, investigation, methodology, and writing of the original draft. HL was responsible for investigation and methodology. ST was responsible for formal analysis, validation, and methodology. CZ was responsible for formal analysis and investigation. JL was responsible for formal analysis and investigation. WT was responsible for formal analysis and investigation. TC was responsible for investigation. YD was responsible for investigation. CW was responsible for formal analysis. DL was responsible for investigation. YA was responsible for resources. WZ was responsible for resources. HW was responsible for resources. KL was responsible for conceptualization and data curation. YF was responsible for data curation. YG was responsible for conceptualization, data curation, and funding acquisition. QH was responsible for conceptualization, data curation, and funding acquisition. XM was responsible for data curation and visualization. BW was responsible for conceptualization, data curation, and funding acquisition. XZ was responsible for conceptualization, resources, supervision, and funding acquisition. YH was responsible for conceptualization, resources, data curation, formal analysis, supervision, funding acquisition, and writing of the original draft.

## Supplementary Material

Supplemental data

Unedited blot and gel images

Supporting data values

## Version 1 02/18/2025

In-Press Preview

## Version 2 04/15/2025

Electronic publication
