# Supplementary material for: Asparagine drives immune evasion in bladder cancer via RIG-I stability and type I IFN signaling
Source: J Clin Invest. 2025 Feb 18;135(8):e186648. doi: 10.1172/JCI186648 (PMC11996873; doi:10.1172/JCI186648)
Supplement: Supplemental data [file jci-135-186648-s080.pdf]

Fig.S1

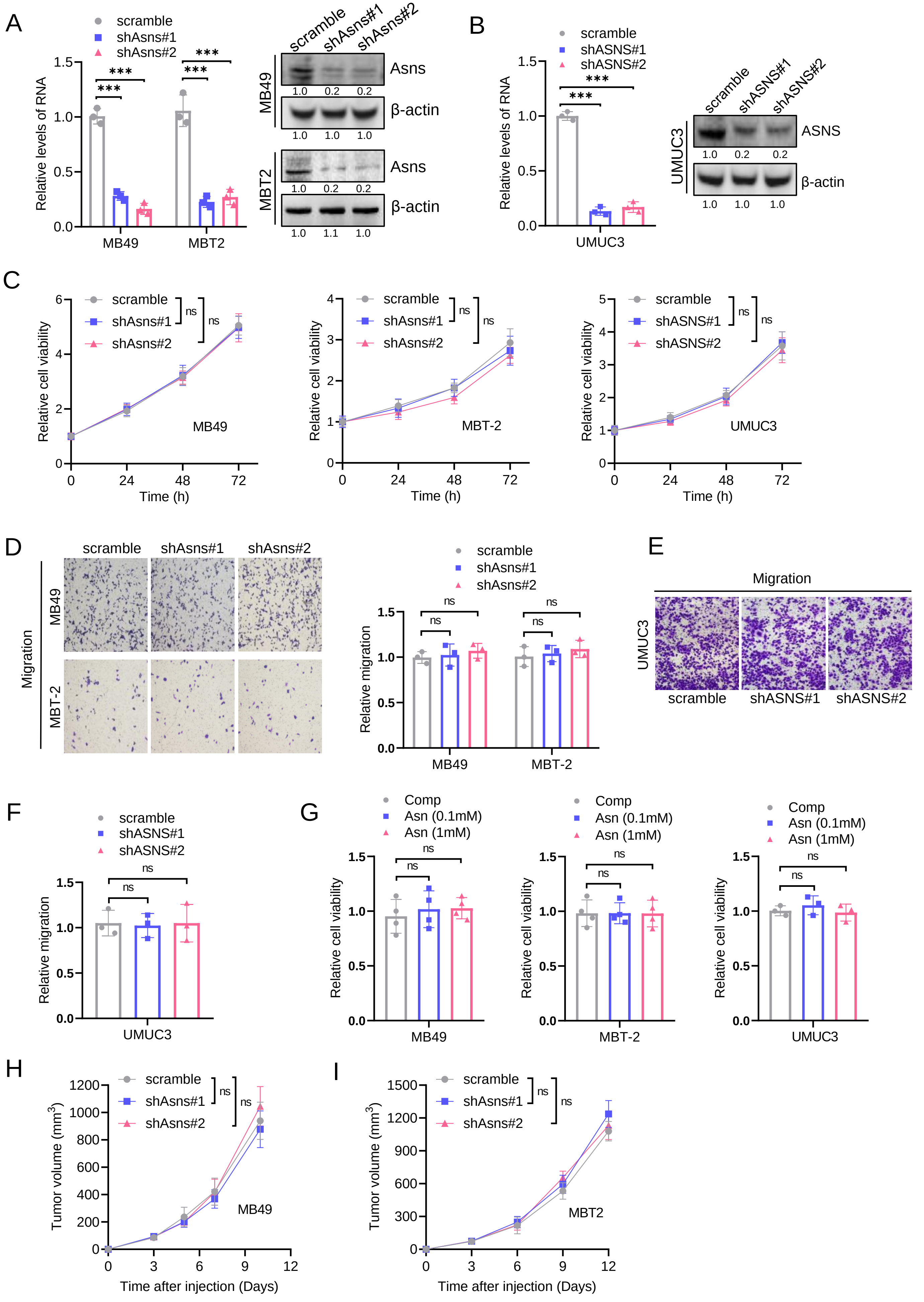
A
scramble
shAsns#1
shAsns#2
Relative levels of RNA
***
MB49
MBT2
scramble shAsns#1 shAsns#2
MB49
Asns
1.0 0.2 0.2
β-actin
1.0 1.0 1.0
MBT2
Asns
1.0 0.2 0.2
β-actin
1.0 1.1 1.0
B
scramble
shASNS#1
shASNS#2
Relative levels of RNA
***
UMUC3
scramble shASNS#1 shASNS#2
UMUC3
ASNS
1.0 0.2 0.2
β-actin
1.0 1.0 1.0
C
scramble
shAsns#1
shAsns#2
ns
Relative cell viability
MB49
MBT-2
shASNS#1
shASNS#2
UMUC3
Time (h)
D
scramble shAsns#1 shAsns#2
Migration
MB49
MBT-2
Relative migration
E
Migration
UMUC3
scramble shASNS#1 shASNS#2
F
Relative migration
G
Comp
Asn (0.1mM)
Asn (1mM)
H
Tumor volume (mm³)
Time after injection (Days)
I
MBT2

Figure S1. ASNS loss has no obvious difference in tumor growth *in vitro* and in immunodeficient nude mice

(**A**) qRT-PCR and western blot analysis of the expression of Asns in murine tumor lines (MB49 and MBT2) stably transfected with scramble, shAsns#1 or shAsns#2.

(**B**) qRT-PCR and western blot analysis of the expression of ASNS in UMUC3 lines stably transfected with scramble, shASNS#1 or shASNS#2.

(**C**) Cell proliferation assay for bladder cancer cells stably transfected with scramble, shASNS#1 or shASNS#2.

(**D-F**) The migration abilities of murine tumor lines (**D**) and UMUC3 cells (**E-F**) with or without ASNS depletion.

(**G**) Cell proliferation assay for bladder cancer cells cultured in complete medium (Comp) and medium added Asn (0.1, 1mM) for 48 h.

(**H**) Tumor growth curves of immunodeficient nude mice (n = 6) injected subcutaneously with scramble or shAsns MB49 cells.

(**I**) Tumor growth curves of immunodeficient nude mice (n = 6) injected subcutaneously with scramble or shAsns MBT2 cells.

Data were mean ± SD. Statistical significance was calculated by one-way ANOVA for A, B, C, D, F, G, H and I. ns, not significant. ***p<0.001.

Fig.S2

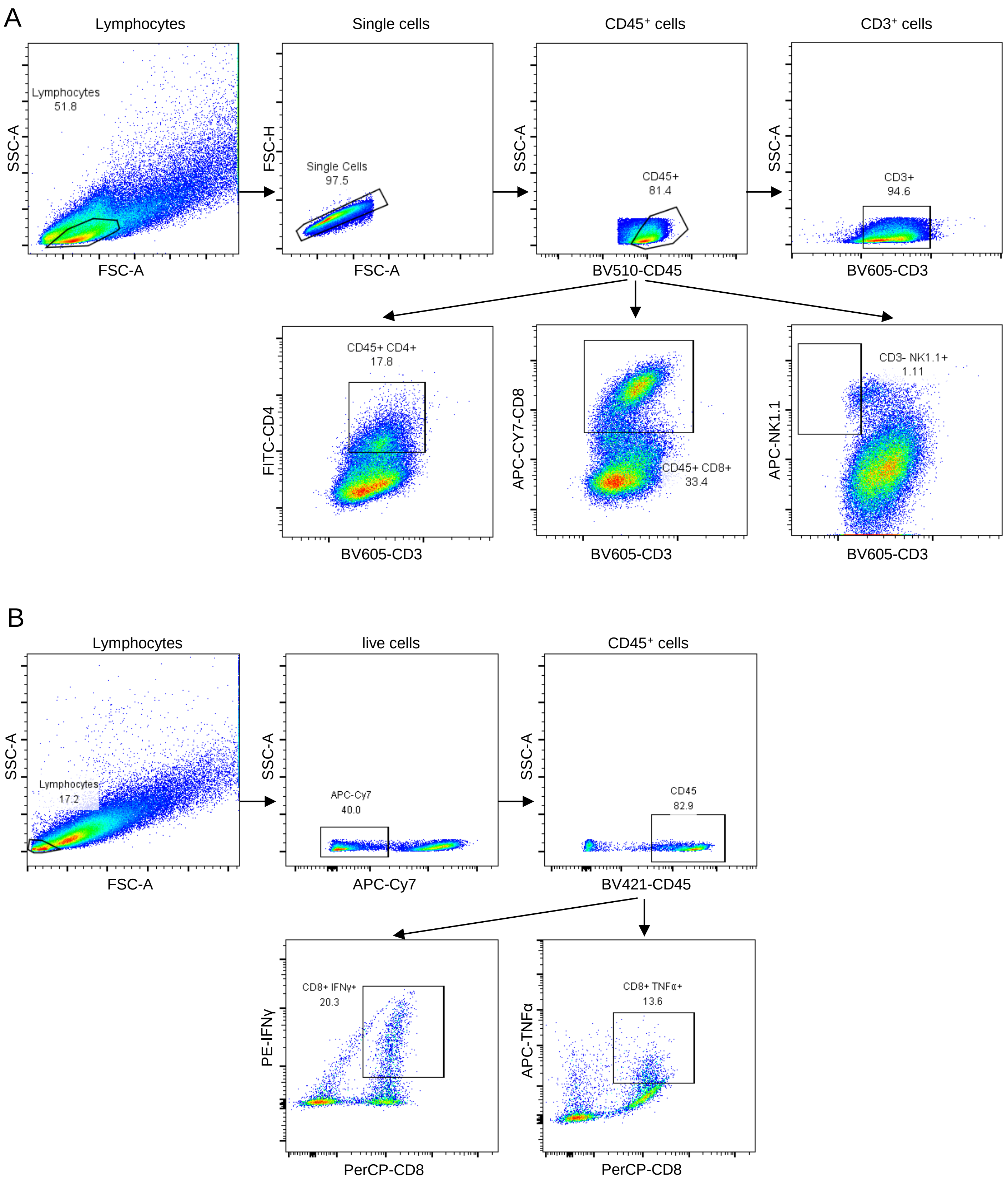

Figure S2. Representative flow cytometry gating strategy

(**A**) Representative flow-cytometry gating strategy for quantifying the number of various immune effector cell subsets in murine tumors.

(**B**) Representative flow cytometry gating strategy for quantifying the number of cytokines secreted by CD8+ T cells in murine tumors.

Fig.S3

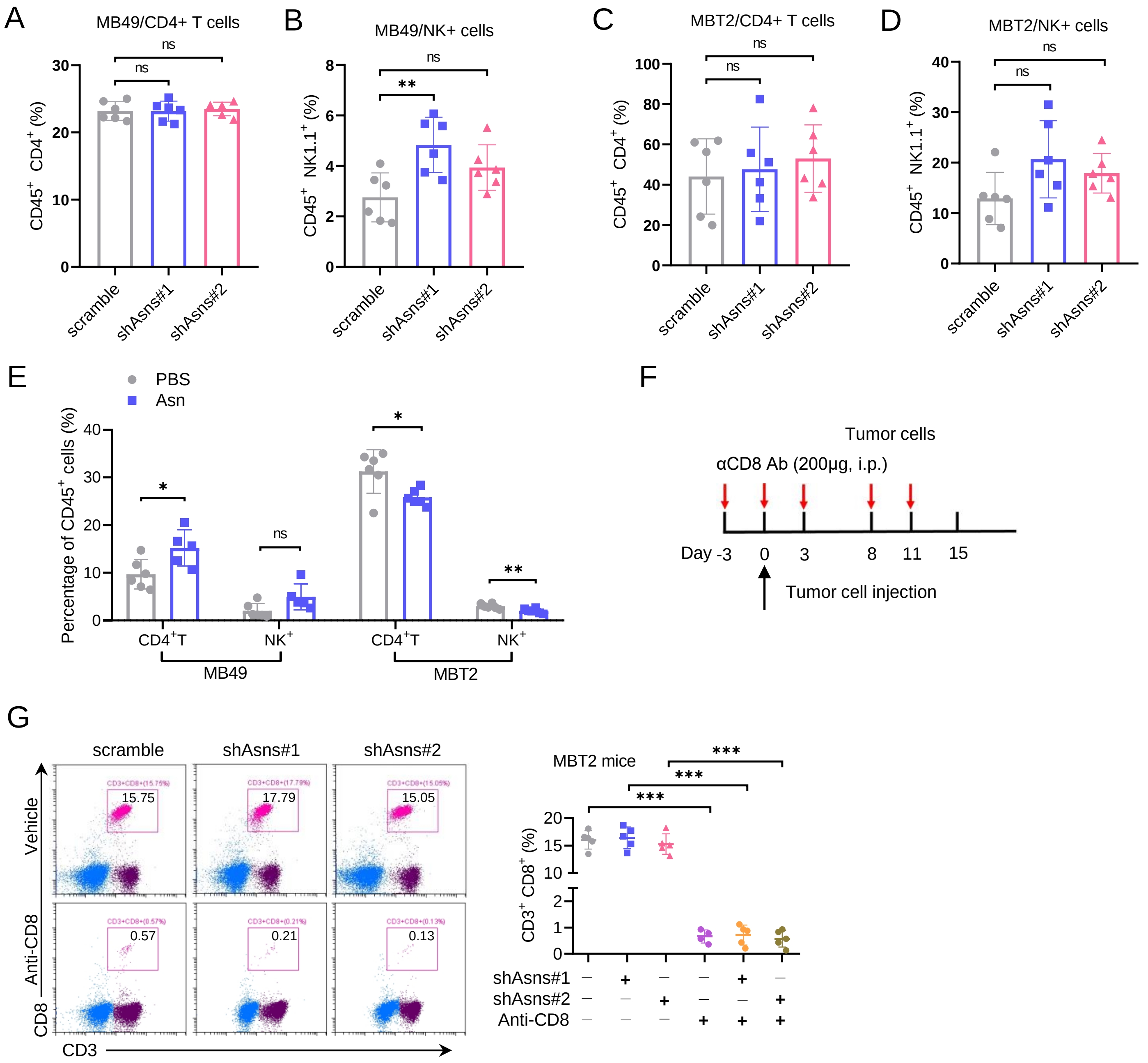
A
MB49/CD4+ T cells
CD45+ CD4+ (%)
scramble
shAsns#1
shAsns#2
ns
B
MB49/NK+ cells
CD45+ NK1.1+ (%)
**
ns
C
MBT2/CD4+ T cells
D
MBT2/NK+ cells
E
PBS
Asn
Percentage of CD45+ cells (%)
CD4+T
NK+
MB49
MBT2
*
**
F
Tumor cells
αCD8 Ab (200μg, i.p.)
Day -3
0
3
8
11
15
Tumor cell injection
G
Vehicle
Anti-CD8
CD8
CD3
15.75
17.79
15.05
0.57
0.21
0.13
MBT2 mice
***
CD3+ CD8+ (%)
shAsns#1
shAsns#2
Anti-CD8

Figure S3. Knockdown of ASNS promotes intratumoral CD8+ T cells infiltration

(**A**) Tumor infiltrating CD4+ T cells from transplanted MB49 tumors (n = 6) in C57BL/6 mice were analyzed by flow cytometry.

(**B**) Tumor infiltrating NK+ cells from transplanted MB49 tumors (n = 6) in C57BL/6 mice were analyzed by flow cytometry.

(**C**) Tumor infiltrating CD4+ T cells from transplanted MBT2 tumors (n = 6) in C3H mice were analyzed by flow cytometry.

(**D**) Tumor infiltrating NK+ cells from transplanted MBT2 tumors (n = 6) in C3H mice were analyzed by flow cytometry.

(**E**) Tumor infiltrating CD4+ T cells and NK cells were analyzed by flow cytometry from transplanted MB49 and MBT2 tumors (n = 6) in syngeneic mice administrated with PBS or Asn.

(**F**) Schematic showed that immunocompetent mice were inoculated with mouse bladder cancer cells and treated with anti-CD8 antibody (200μg per mice) on day -3, 0, 3, 8, and 11.

(**G**) C3H mice were subcutaneously inoculated with indicated MBT2 tumor cells and treated with anti-CD8 antibody. Flow cytometry analysis of CD8+ T cells content in peripheral blood of mice (n = 5) at the end of experiment.

Data were mean ± SD. Statistical significance was calculated by two tailed unpaired Student's t-tests for E. One-way ANOVA for A, B, C and D. Two-way ANOVA for G. ns, not significant. *p<0.05, **p<0.01, ***p<0.001.

Fig.S4

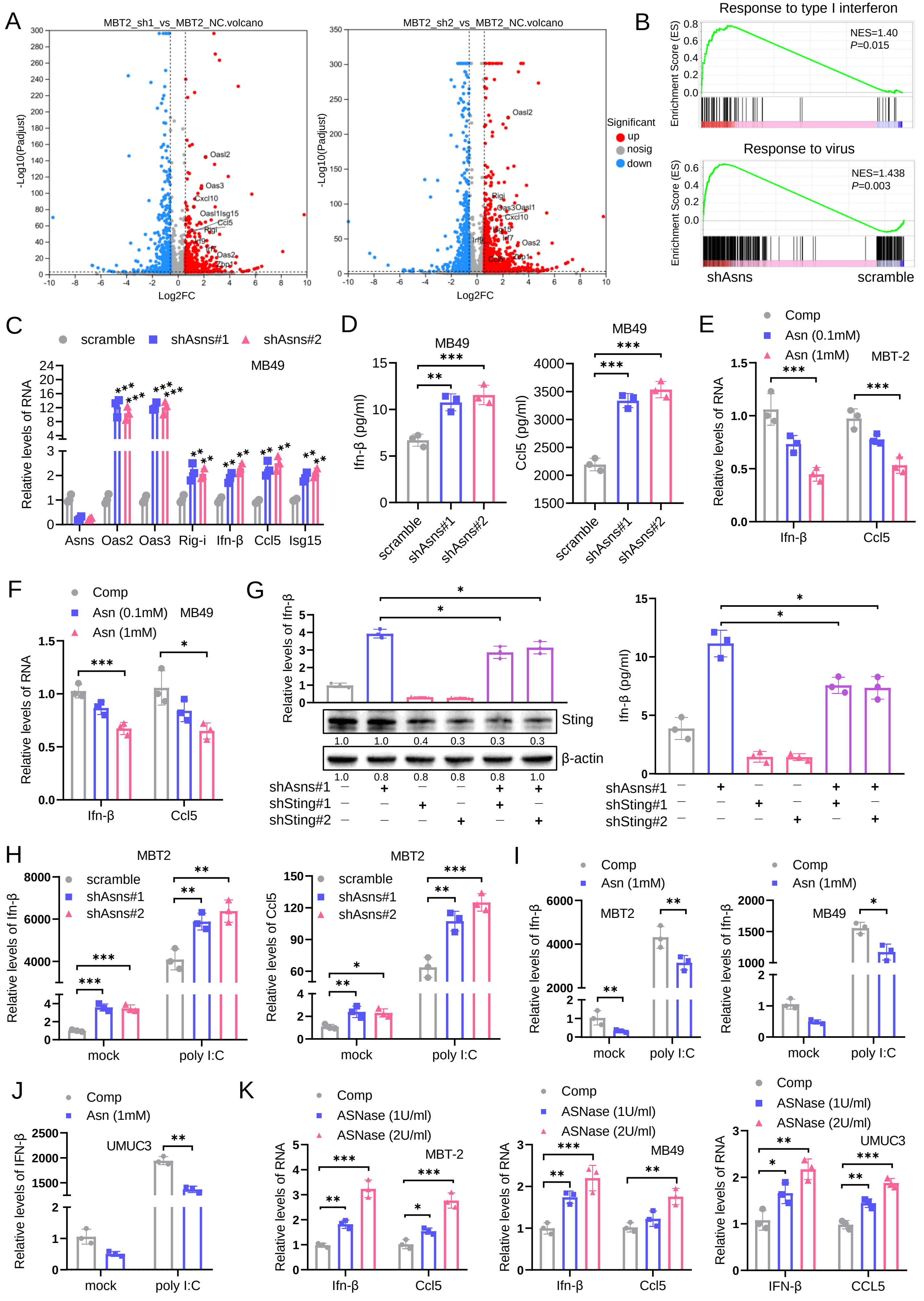

A
MBT2_sh1_vs_MBT2_NC.volcano
MBT2_sh2_vs_MBT2_NC.volcano
-Log10(Padjust)
Log2FC
Oasl2
Oas3
Cxcl10
Oasl1Isg15
Ccl5
Rigi
Irf9
Irf7
Oas2
Zbp1
Rigi
Oas3Oasl1
Cxcl10
Isg15
Irf7
Irf9
Oas2
Zbp1
Ccl5
Significant
up
nosig
down
B
Response to type I interferon
NES=1.40
P=0.015
Response to virus
NES=1.438
P=0.003
Enrichment Score (ES)
shAsns
scramble
C
scramble
shAsns#1
shAsns#2
MB49
Relative levels of RNA
Asns
Oas2
Oas3
Rig-i
Ifn-β
Ccl5
Isg15
D
MB49
Ifn-β (pg/ml)
Ccl5 (pg/ml)
scramble
shAsns#1
shAsns#2
E
Comp
Asn (0.1mM)
Asn (1mM)
MBT-2
Relative levels of RNA
Ifn-β
Ccl5
F
Comp
Asn (0.1mM)
MB49
Asn (1mM)
Relative levels of RNA
Ifn-β
Ccl5
G
Relative levels of Ifn-β
Sting
β-actin
1.0 1.0 0.4 0.3 0.3 0.3
1.0 0.8 0.8 0.8 0.8 1.0
shAsns#1
shSting#1
shSting#2
Ifn-β (pg/ml)
H
MBT2
scramble
shAsns#1
shAsns#2
Relative levels of Ifn-β
Relative levels of Ccl5
mock
poly I:C
I
Comp
Asn (1mM)
MBT2
MB49
Relative levels of Ifn-β
mock
poly I:C
J
Comp
Asn (1mM)
UMUC3
Relative levels of IFN-β
mock
poly I:C
K
Comp
ASNase (1U/ml)
ASNase (2U/ml)
MBT-2
MB49
UMUC3
Relative levels of RNA
Ifn-β
Ccl5
IFN-β
CCL5

Figure S4. Depletion of ASNS activates IFN-I signaling pathway

(**A**) Volcano plot showing differentially expressed genes by Asns loss in RNA-seq analysis.

(**B**) GSEA of the indicated gene signatures between scramble and shAsns-deficient MBT2 cells.

(**C**) qRT-PCR showed the relative expression levels of ISGs genes in the indicated MB49 cells.

(**D**) ELISA experiment revealed the expression levels of Ifn-β and Ccl5 in culture supernatants of the indicated MB49 cells.

(**E-F**) qRT-PCR assay showed the expression levels of Ifn-β and Ccl5 in MBT2 (**E**) and MB49 (**F**) cells cultured in complete medium (Comp) and medium added Asn for 48 h.

(**G**) qRT-PCR and ELISA assays showed the expression levels of Ifn-β in MBT2 cells stably transfected with scramble or shAsns#1, and those co-transfected with shSting#1 or shSting#2. Western blot assay showed the expression levels of STING proteins in the indicated.

(**H**) Scramble or shAsns MBT2 cells were transfected with poly (I:C) (2μg/ml) for 8 h and the mRNA levels of Ifn-β and Ccl5 were determined by qRT-PCR.

(**I**) The expression levels of Ifn-β mRNA were determined by qRT-PCR in the indicated MBT2 and MB49 cells .

(**J**) The expression levels of IFN-β mRNA were determined by qRT-PCR in the indicated UMUC3 cells.

(**K**) qRT-PCR assay showed the expression levels of IFN-β and CCL5 in bladder cancer cells treated with ASNase for 48 h.

Data were mean ± SD. Statistical significance was calculated by two tailed unpaired Student's t-tests for I and J. One-way ANOVA for C, D, E, F, H and K. Two-way ANOVA for G. *$p<0.05$, **$p<0.01$, ***$p<0.001$.

Fig.S5

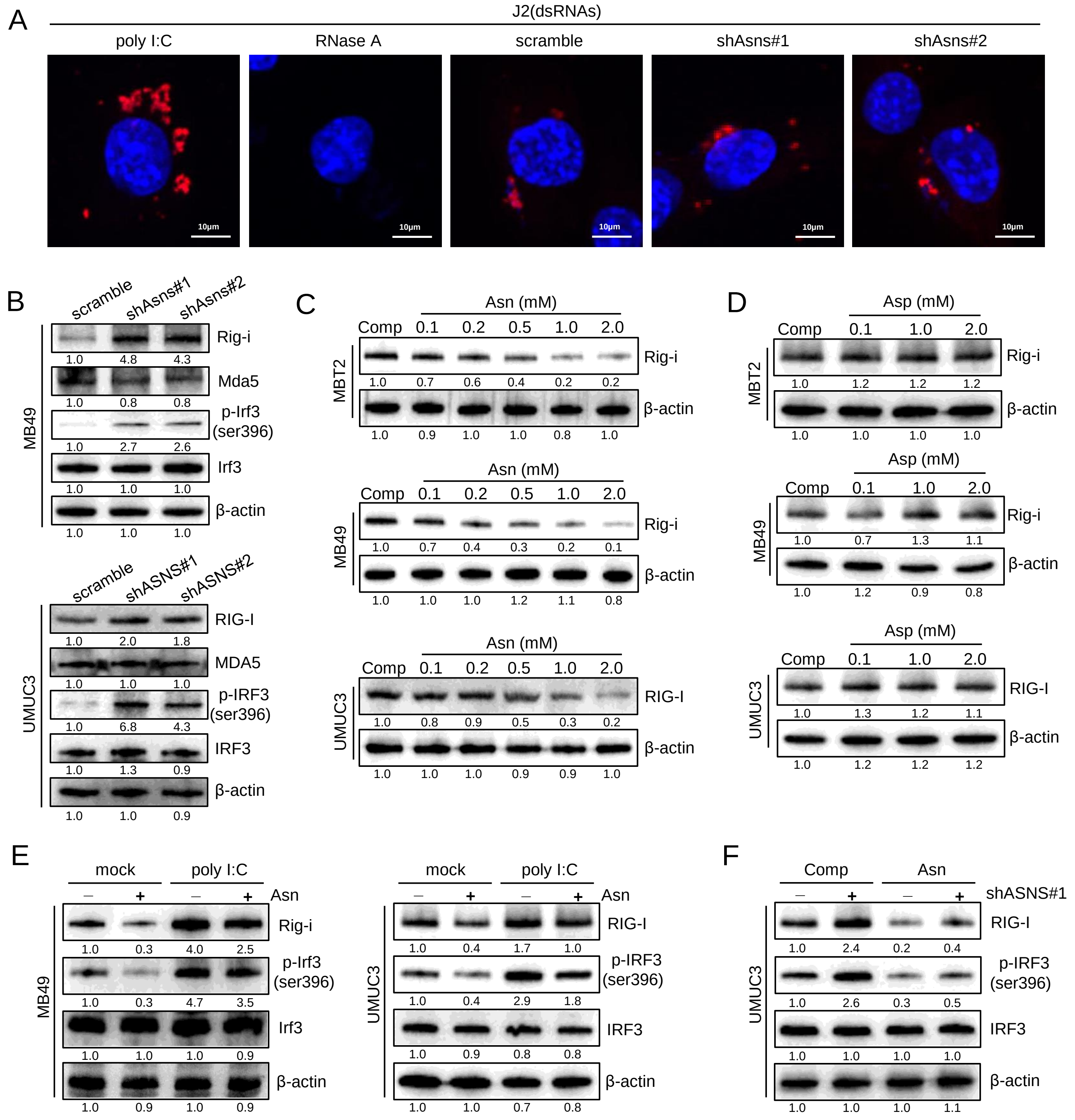
A
J2(dsRNAs)
poly I:C
RNase A
scramble
shAsns#1
shAsns#2
10μm
B
scramble
shAsns#1
shAsns#2
MB49
Rig-i
1.0 4.8 4.3
Mda5
1.0 0.8 0.8
p-Irf3 (ser396)
1.0 2.7 2.6
Irf3
1.0 1.0 1.0
β-actin
1.0 1.0 1.0
scramble
shASNS#1
shASNS#2
UMUC3
RIG-I
1.0 2.0 1.8
MDA5
1.0 1.0 1.0
p-IRF3 (ser396)
1.0 6.8 4.3
IRF3
1.0 1.3 0.9
β-actin
1.0 1.0 0.9
C
Asn (mM)
Comp 0.1 0.2 0.5 1.0 2.0
MBT2
Rig-i
1.0 0.7 0.6 0.4 0.2 0.2
β-actin
1.0 0.9 1.0 1.0 0.8 1.0
Asn (mM)
Comp 0.1 0.2 0.5 1.0 2.0
MB49
Rig-i
1.0 0.7 0.4 0.3 0.2 0.1
β-actin
1.0 1.0 1.0 1.2 1.1 0.8
Asn (mM)
Comp 0.1 0.2 0.5 1.0 2.0
UMUC3
RIG-I
1.0 0.8 0.9 0.5 0.3 0.2
β-actin
1.0 1.0 1.0 0.9 0.9 1.0
D
Asp (mM)
Comp 0.1 1.0 2.0
MBT2
Rig-i
1.0 1.2 1.2 1.2
β-actin
1.0 1.0 1.0 1.0
Asp (mM)
Comp 0.1 1.0 2.0
MB49
Rig-i
1.0 0.7 1.3 1.1
β-actin
1.0 1.2 0.9 0.8
Asp (mM)
Comp 0.1 1.0 2.0
UMUC3
RIG-I
1.0 1.3 1.2 1.1
β-actin
1.0 1.2 1.2 1.2
E
mock
poly I:C
– + – + Asn
MB49
Rig-i
1.0 0.3 4.0 2.5
p-Irf3 (ser396)
1.0 0.3 4.7 3.5
Irf3
1.0 1.0 1.0 0.9
β-actin
1.0 0.9 1.0 0.9
mock
poly I:C
– + – + Asn
UMUC3
RIG-I
1.0 0.4 1.7 1.0
p-IRF3 (ser396)
1.0 0.4 2.9 1.8
IRF3
1.0 0.9 0.8 0.8
β-actin
1.0 1.0 0.7 0.8
F
Comp
Asn
– + – + shASNS#1
UMUC3
RIG-I
1.0 2.4 0.2 0.4
p-IRF3 (ser396)
1.0 2.6 0.3 0.5
IRF3
1.0 1.0 1.0 1.0
β-actin
1.0 1.0 1.0 1.1

Figure S5. Asparagine suppresses the expression of RIG-I protein

(**A**) Endogenous dsRNAs accumulation in the indicated MBT2 cells was tested by IF with an anti-dsRNA specific J2 antibody. Scale bars, 10 μm.

(**B**) Western blot showed the indicated proteins in MB49 and UMUC3 cells stably transfected with scramble, shASNS#1 or shASNS#2.

(**C**) Western blot showed the expression of RIG-I in bladder cancer cells treated with different concentrations of Asn for 48 h.

(**D**) Western blot showed the expression of RIG-I in bladder cancer cells treated with different concentrations of Asp for 48 h.

(**E**) MB49 and UMUC3 cells were cultured in complete medium (Comp) and medium added Asn (1mM) for 48 h and then were transfected with poly (I:C) (2μg/ml) for 8 h. Western blot showed the indicated proteins in above samples.

(**F**) Western blot showed the indicated proteins in UMUC3 cells stably transfected with scramble and shASNS#1, and then were cultured in complete medium (Comp) and medium added Asn (1mM) for 48 h.

Fig.S6

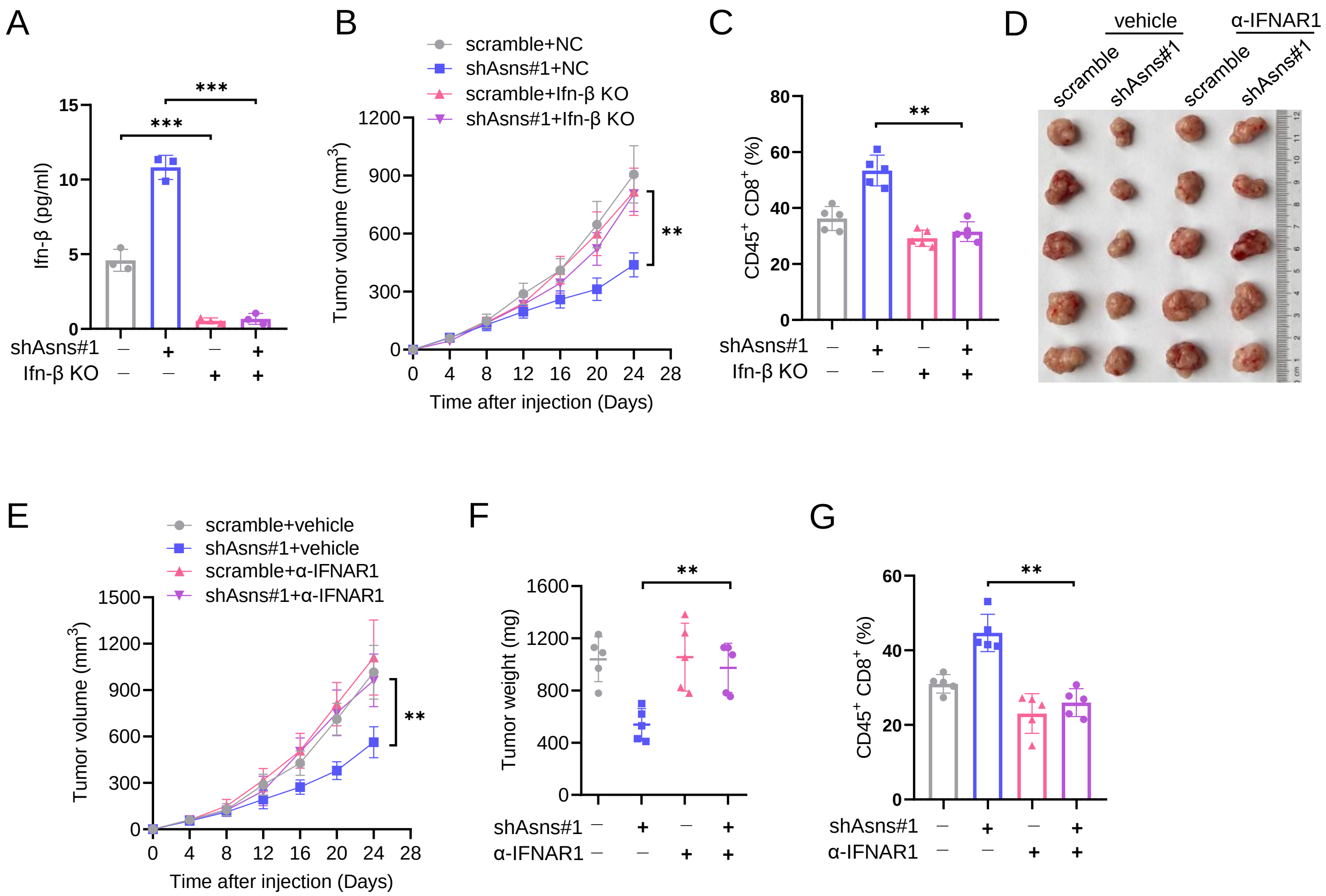

Figure S6. IFN-β deletion rescues the tumor growth inhibition mediated by ASNS knockdown

(**A**) ELISA assay showed the expression levels of Ifn-β in MB49 cells in the indicated group.

(**B**) Tumor growth curves of immunocompetent C57BL/6 mice (n = 5) injected subcutaneously with indicated MB49 cells.

(**C**) Tumor infiltrating CD8+ T cells from transplanted MB49 tumors (n = 5) in C57BL/6 mice were analyzed by flow cytometry.

(**D-F**) Tumor growth and tumor weight in immunocompetent C57BL/6 mice (n = 5) injected subcutaneously with either the scramble- or Asns-ablated MB49 cells and treated with 200 μg of anti-IFNAR1 or isotype control on days 0, 7, 14, and 21.

(**G**) Tumor infiltrating CD8+ T cells in MB49 tumors (n = 5) of indicated groups were analyzed by flow cytometry.

Data were mean ± SD. Statistical significance was calculated by two-way ANOVA for A, B, C, E, F and G. **p<0.01, ***p<0.001.

Fig.S7

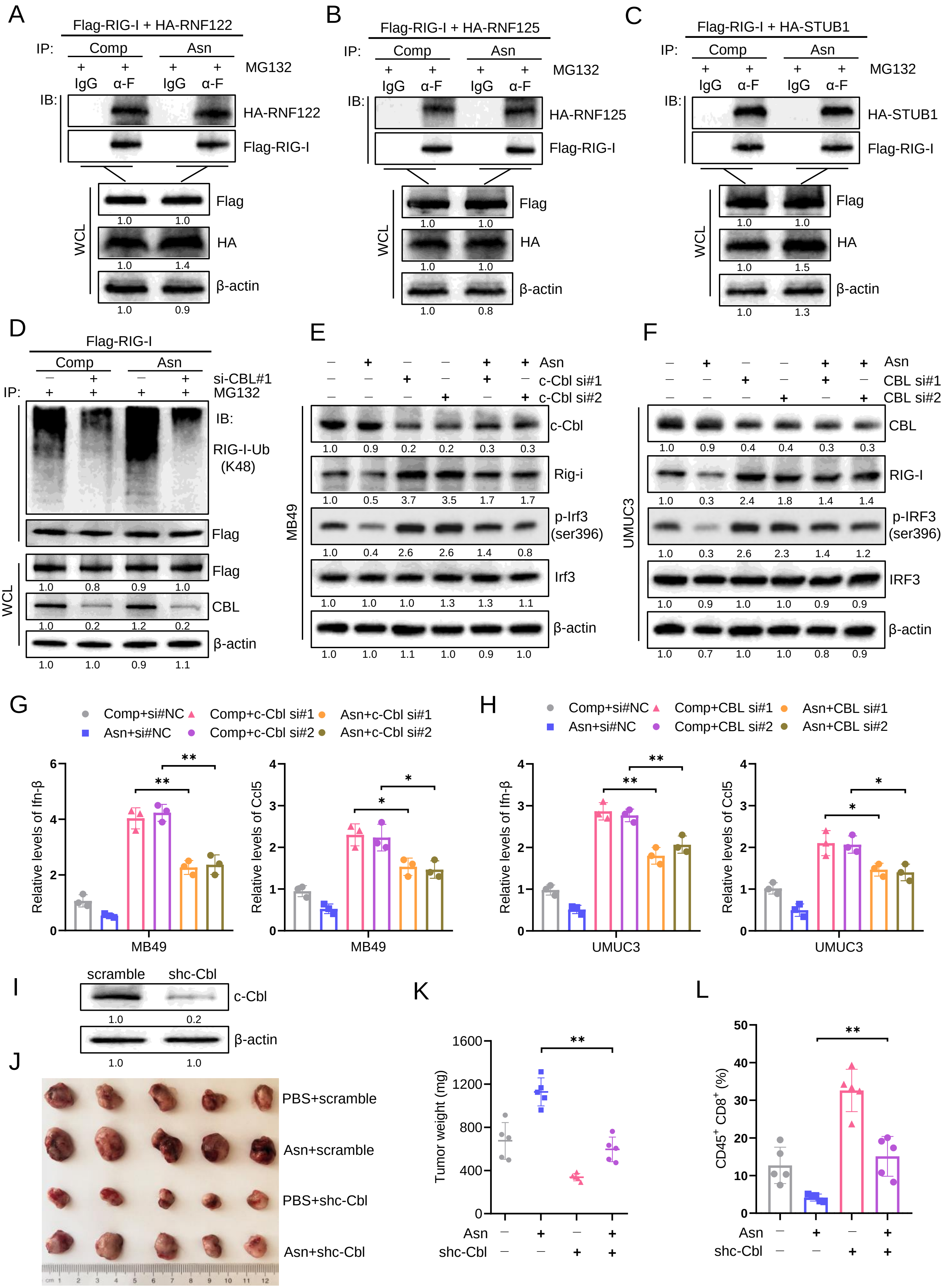
A
Flag-RIG-I + HA-RNF122
IP: Comp Asn
MG132
IgG α-F IgG α-F
IB:
HA-RNF122
Flag-RIG-I
WCL
Flag
HA
β-actin
B
Flag-RIG-I + HA-RNF125
HA-RNF125
C
Flag-RIG-I + HA-STUB1
HA-STUB1
D
Flag-RIG-I
si-CBL#1
IB: RIG-I-Ub (K48)
CBL
E
Asn
c-Cbl si#1
c-Cbl si#2
MB49
c-Cbl
Rig-i
p-Irf3 (ser396)
Irf3
F
CBL si#1
CBL si#2
UMUC3
CBL
RIG-I
p-IRF3 (ser396)
IRF3
G
Comp+si#NC
Asn+si#NC
Comp+c-Cbl si#1
Comp+c-Cbl si#2
Asn+c-Cbl si#1
Asn+c-Cbl si#2
Relative levels of Ifn-β
Relative levels of Ccl5
MB49
H
Comp+CBL si#1
Comp+CBL si#2
Asn+CBL si#1
Asn+CBL si#2
UMUC3
I
scramble shc-Cbl
c-Cbl
β-actin
J
PBS+scramble
Asn+scramble
PBS+shc-Cbl
Asn+shc-Cbl
K
Tumor weight (mg)
Asn
shc-Cbl
L
CD45+ CD8+ (%)

Figure S7. Asparagine inhibits the expression of RIG-I through facilitating its combination with E3 ligase CBL

(**A**) HEK293T cells were transfected with Flag-RIG-I and HA-RNF122 and cultured in complete medium (Comp) and medium added Asn (1mM) for 48 h, followed by co-immunoprecipitation and immunoblotting analysis with the indicated antibodies.

(**B**) HEK293T cells were transfected with Flag-RIG-I and HA-RNF125 and cultured in complete medium (Comp) and medium added Asn (1mM) for 48 h, followed by co-immunoprecipitation and immunoblotting analysis with the indicated antibodies.

(**C**) HEK293T cells were transfected with Flag-RIG-I and HA-STUB1 and cultured in complete medium (Comp) and medium added Asn (1mM) for 48 h, followed by co-immunoprecipitation and immunoblotting analysis with the indicated antibodies.

(**D**) Flag-RIG-I-overexpressed HEK293T cells were cultured in complete medium (Comp) and medium added Asn (1mM), and those co-transfected with si-NC and si-CBL#1 for 48 h, followed by co-immunoprecipitation and immunoblotting analysis with the indicated antibodies.

(**E**) Western blot analysis of cell lysates from the indicated MB49 cells.

(**F**) Western blot analysis of cell lysates from the indicated UMUC3 cells.

(**G**) qRT-PCR revealed the expression levels of Ifn-β and Ccl5 in the indicated MB49.

(**H**) qRT-PCR revealed the expression levels of IFN-β and CCL5 in the indicated UMUC3 cells.

(**I**) Western blot analysis of cell lysates from the indicated MBT2 cells.

(**J-K**) Tumor image and tumor weight of immunocompetent C3H mice (n = 5) injected subcutaneously with indicated MBT2 cells.

(**L**) Tumor infiltrating CD8+ T cells from transplanted MBT2 tumors (n = 5) in C3H mice were analyzed by flow cytometry.

Data were mean ± SD. Statistical significance was calculated by two-way ANOVA for G, H, K and L. $^{*}p<0.05$, $^{**}p<0.01$.

Fig.S8

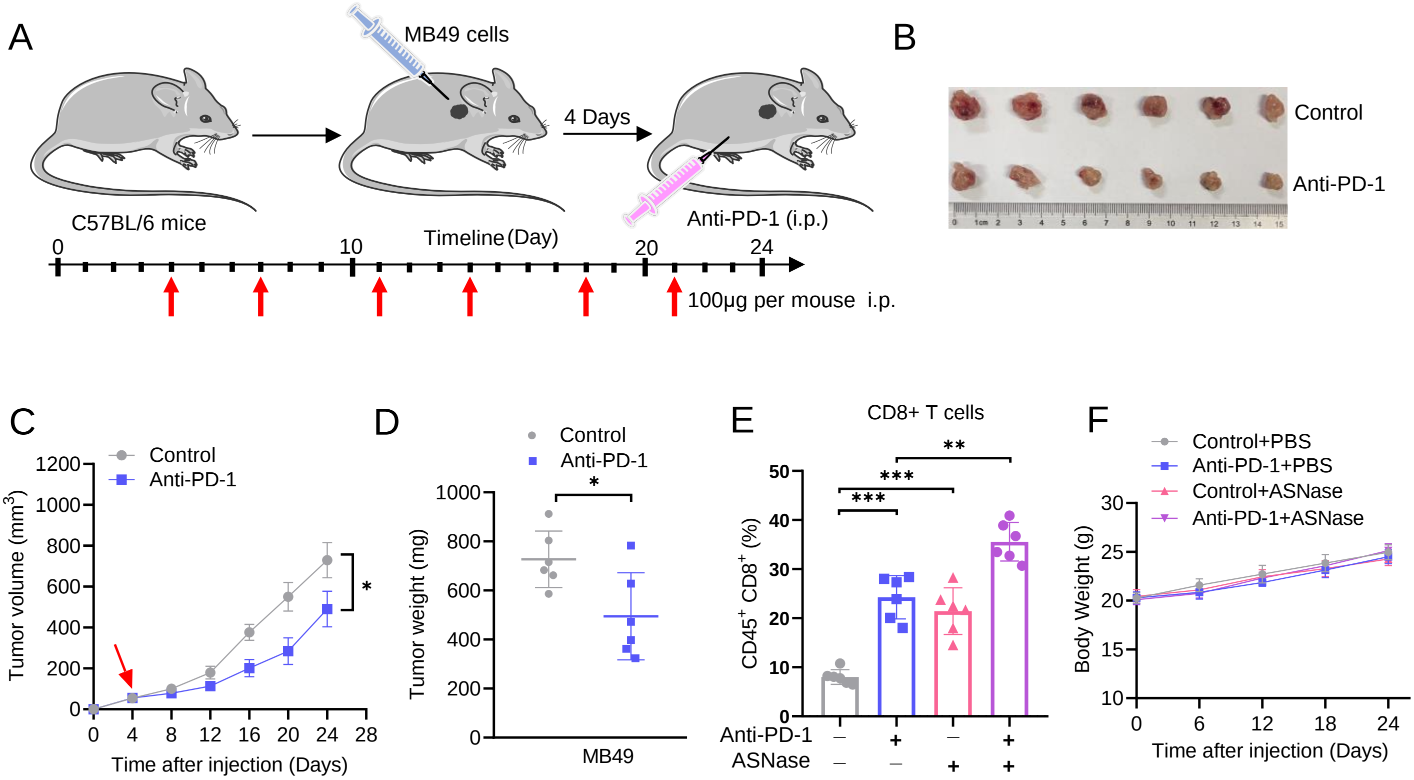

Figure S8. ASNase treatment enhances the efficacy of PD-1 blockade in bladder cancer

(**A**) Schematic of anti-PD-1 antibody administration.

(**B-D**) Tumor growth (**B** and **C**) and tumor weight (**D**) in immunocompetent C57BL/6 mice injected subcutaneously with MB49 cells, and treated with anti-PD-1 or isotype control (n = 6).

(**E**) Tumor infiltrating CD8+ T cells in MBT2 tumors of indicated groups were analyzed by flow cytometry.

(**F**) The body weights among different groups during experimental procedure.

Data were mean ± SD. Statistical significance was calculated by two tailed unpaired Student's t-tests for C and D. Two-way ANOVA for E and F. *$p<0.05$, **$p<0.01$, ***$p<0.001$.

Fig.S9

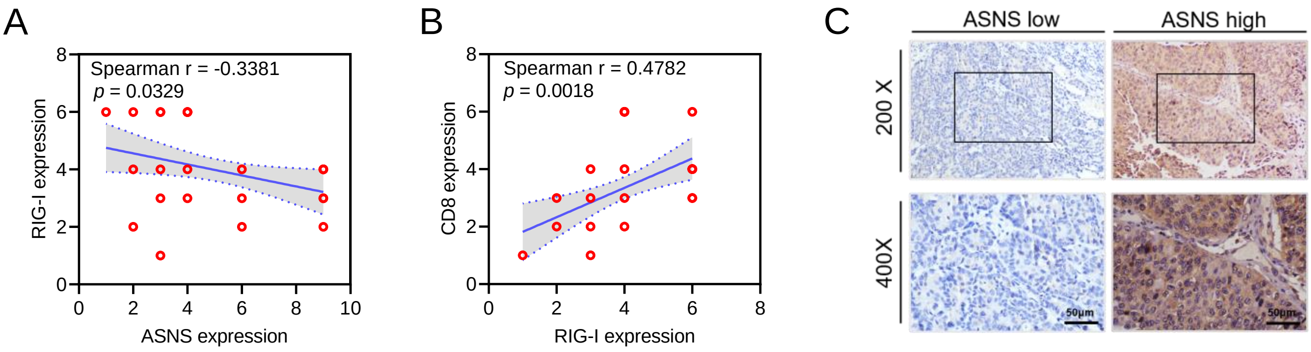

Figure S9. The correlation between ASNS, RIG-I and CD8 expression in bladder cancer samples

(**A**) Correlation analysis of ASNS and RIG-I expression in bladder cancer clinical samples (n = 40).

(**B**) Correlation analysis of RIG-I and CD8 expression in bladder cancer clinical samples (n = 40).

(**C**) Representative immunohistochemical image of low and high ASNS expression patient in 301-immune cohort. Scale bar, 50 μm.

Supplemental table 1: The sequences of primers and oligonucleotides used in this study.	
Primers for PCR（5’-3'）	
ASNS F	ACAGTTCGTGCTTCAGTAGGT
ASNS R	GGGACTCTCAGTTCAAGACCAT
ACTB F	TGGCACCCAGCACAATGAA
ACTB R	CTAAGTCATAGTCCGCCTAGAAGCA
Asns F	TGCGTCTGTGGAAATGGTCAA
Asns R	CTCTAGGTCAAAGCCTGGGAA
18S F	TAAACGATGCCGACTGGCGA
18S R	CAAATTAAGCCGCAGGCCCA
Oas2 F	CCTTGGAAAGTGCCAGTACCT
Oas2 R	TTGCCAGATCACTCCAGAAGC
Oas3 F	CAAGACCATCGGTGACTTCCT
Oas3 R	ACTTCACACAGCGGCCTTTAC
Rig-i F	TTTGGATGCCCTGTACCATGC
Rig-i R	CCCTTTAGTGTCTCGGATCTGTC
Ifn-β F	CAGCTCCAAGAAAGGACGAAC
Ifn-β R	GGCAGTGTAACTCTTCTGCAT
Ccl5 F	ATATGGCTCGGACACCACT
Ccl5 R	CTTCGAGTGACAAACACGACTG
IFN-β F	ATGACCAACAAGTGTCTCCTCC
IFN-β R	GGAATCCAAGCAAGTTGTAGCTC
CCL5 F	CCATATTCCTCGGACACCACA
CCL5 R	TTCGGGTGACAAAGACGACTG
shRNA sequence	
shASNS#1	CCGGGCTGTATGTTCAGAAGCTAAACTCGAGTTTAGCTTCTGAACATACAGCTTTTTG
shASNS#2	CCGGGGATGGTGAGATAATCCTTCACTCGAGTGAAGGATTATCTCACCATCCTTTTTG
shAsns#1	CCGGGCCAGATATGAGAATTCCAAACTCGAGTTTGGAATTCTCATATCTGGCTTTTTG
shAsns#2	CCGGGGAAATGGTCAAATACCATCACTCGAGTGATGGTATTTGACCATTTCCTTTTTG
shRig-i#1	CCGGGCAAAGATATTCTGCGCCAAACTCGAGTTTGGCGCAGAATATCTTTGCTTTTTG
shMavs#1	CCGGCCAGTGCTGATCTATTAGGAACTCGAGTTCCTAATAGATCAGCACTGGTTTTTG
shMAVS#1	CCGGCCTTACTTTATCTTGTGCCTTCTCGAGAAGGCACAAGATAAAGTAAGGTTTTTG
shCbl	CCGGGCGCACTGTCTTGTCAAGATATCTCGAGATATCTTGACAAGACAGTGCGCTTTTTG
sgRNA sequence	
sgIfnb	GGCGGACTTCAAGATCCCTA
siRNA sequence	
c-Cbl#1-S	CGCCUUAUAUCUUAGACCUTT
c-Cbl#1-AS	AGGUCUAAGAUAUAAGGCGTT
c-Cbl#2-S	GGAGACACUUUCCGGAUUATT
c-Cbl#2-AS	UAAUCCGGAAAGUGUCUCCTT
CBL#1-S	GGACACCUCAUGUGCACAUTT
CBL#1-AS	AUGUGCACAUGAGGUGUCCTT
CBL#2-S	CCGGGAAUUUGUUUCCAUUTT
CBL#2-AS	AAUGGAAACAAAUUCCCGGTT

Supplemental table 2: Multiple cytokines and chemokines between Asns knockdown and the control groups in MBT2 cells culture supernatants.

gene	NC1	NC2	NC3	Sh1- 1	Sh1- 2	Sh1- 3	Sh2-1	Sh2-2	Sh2-3
CXCL13	16.93	16.93	16.93	12.17	9.51	9.51	9.51	9.51	12.17
CCL27	314.77	250.79	330.3	183	165.24	217.47	200.4	217.47	183
CXCL5	3476.95	3524.78	3203.52	4406.86	4432.56	4613.93	3547.1	3336.43	4076.03
CCL24	0.015	0.015	0.015	7.94	0.015	0.015	7.94	0.015	1.49
CCL11	13.71	13.32	18.5	4.14	3.68	4.09	8.61	6.51	6.6
CX3CL1	48.99	45.5	48.99	45.5	37.93	37.93	33.75	41.83	37.93
GM-CSF	52.67	58.49	59.08	35.92	41.54	40.1	44.57	41.9	47.1
CCL1	0.015	0.015	0.86	0.015	0.015	0.64	0.015	0.35	0.64
CXCL11	0.015	0.015	0.015	0.015	0.015	0.015	0.015	147.76	0.015
IFN-γ	0.015	0.015	0.015	0.015	0.015	0.015	0.015	0.015	0.015
IL-10	0.015	0.015	25.98	25.98	0.015	25.98	0.015	0.015	0.015
IL-16	93.04	81.01	81.01	64.15	52.55	43.83	60.44	67.73	43.83
IL-1b	11.32	7.93	7.93	0.015	0.015	0.015	0.015	0.015	0.015
IL-2	0.34	0.51	0.68	0.22	0.1	0.1	0.1	0.015	0.015
IL-4	0.015	0.015	0.015	5.87	0.015	0.015	0.015	0.015	0.015
IL-6	47.31	49.52	50.26	61.18	58.83	61.54	95.09	94.38	98.98
CXCL10	1488.11	1356.13	1283.43	2141.7	1395.62	1156.75	1182.71	1208.35	1551.91
CXCL1	2816.85	2624.07	2969.6	2257.09	1890.61	1764.69	1553.92	1714.79	1981.86
CCL2	10479.99	10639.22	10794.17	8093.24	8764.71	9372.2	10074.73	9787.22	11755.48
CCL7	421.24	447.39	489.7	432.65	452.18	466.15	626.8	573.39	586.06
CCL12	0.015	0.03	0.03	0.03	0.03	0.015	0.03	0.015	0.015
CCL22	2.08	2.43	2.75	1.26	1.89	3.64	3.64	3.06	3.21
CCL3	7.23	8.6	7.98	4.75	5.46	6.36	9.72	7.23	7.45
CCL4	129.93	123.15	145.4	135.63	150.42	160.3	119.21	96.46	104.98
CCL20	41.85	41.51	47.29	160.72	142.24	119.65	43.75	35.62	44.81
CCL19	138.1	95.11	118.76	95.11	60.09	95.11	60.09	95.11	60.09
CCL5	1010.75	1007.83	1430.1	3040.51	3109.44	3606.44	3805.81	2278.37	2547.28
CXCL16	1.97	2.26	1.65	1.17	0.015	0.37	0.05	0.37	0.9
CXCL12	68.31	68.31	68.31	55.07	25.17	47.18	37.77	37.77	47.18
CCL17	20.8	27.5	27.5	27.5	20.8	26.12	22.11	28.89	30.3
TNF-a	29.02	17.14	21.19	12.94	17.14	29.02	32.83	29.02	12.94
