# Supplementary material for: Asparagine drives immune evasion in bladder cancer via RIG-I stability and type I IFN signaling
Source: J Clin Invest. 2025 Feb 18;135(8):e186648. doi: 10.1172/JCI186648 (PMC11996873; doi:10.1172/JCI186648)

Figure 3G

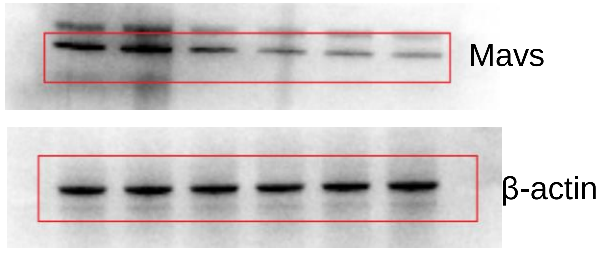

Figure 3L

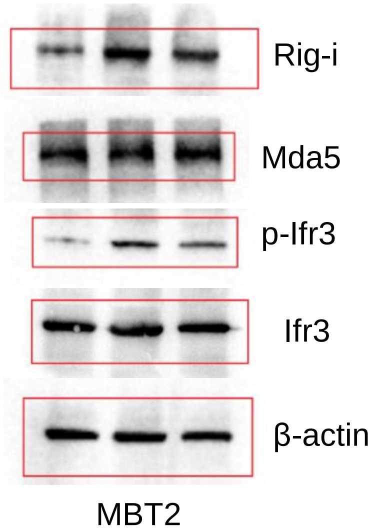

Figure 3M

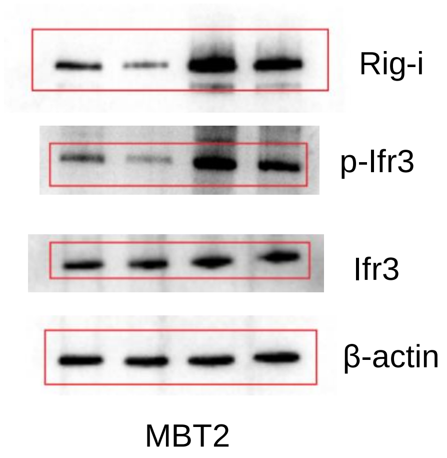

Figure 3N

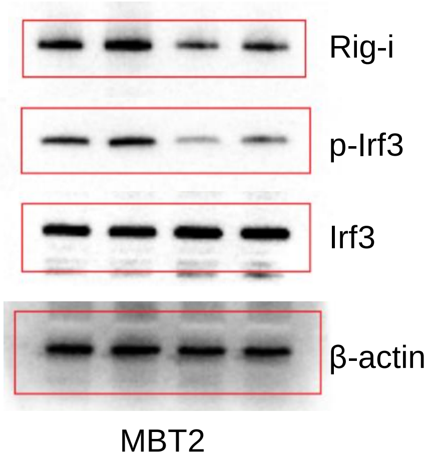

Figure 4A

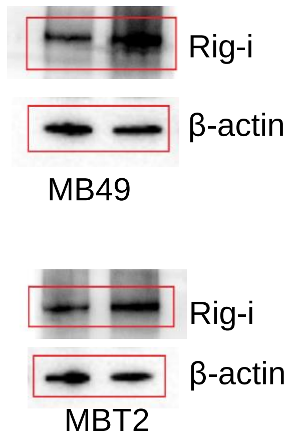

Figure 4B

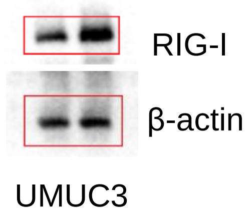

Figure 4C

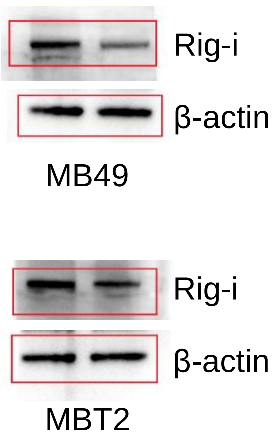

Figure 4D

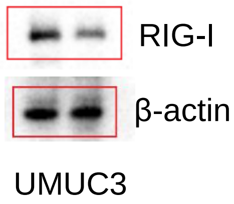

Figure 4E

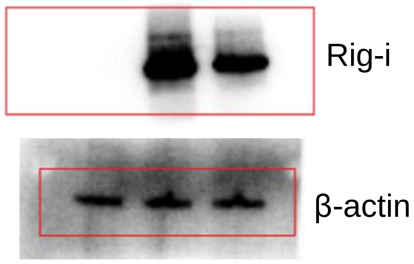

Figure 4F

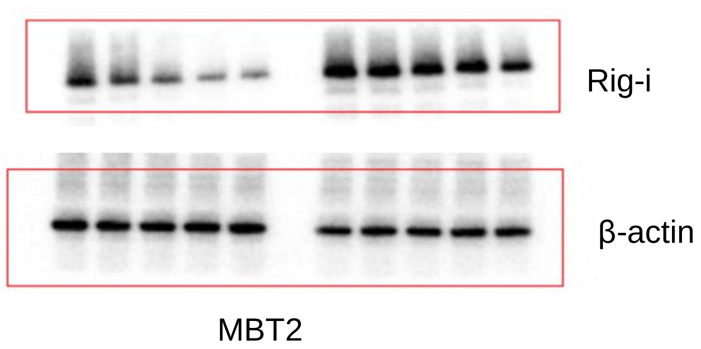

Figure 4I

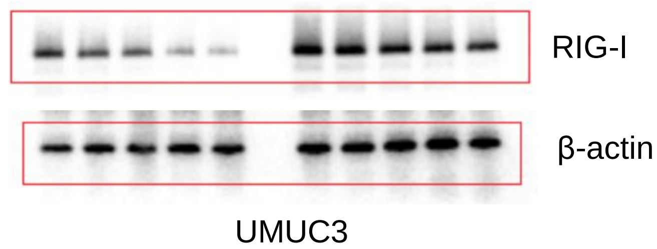

Figure 4J

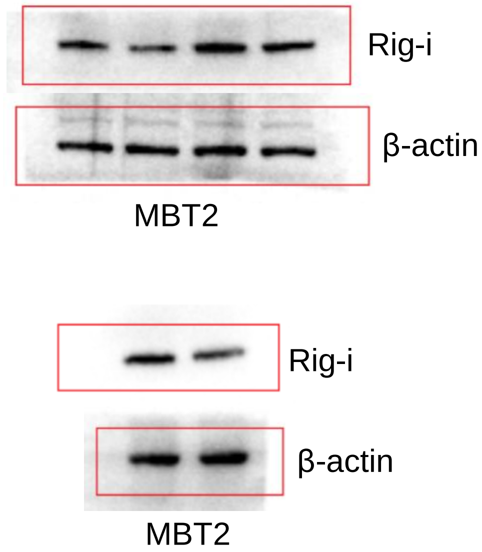

Figure 4K

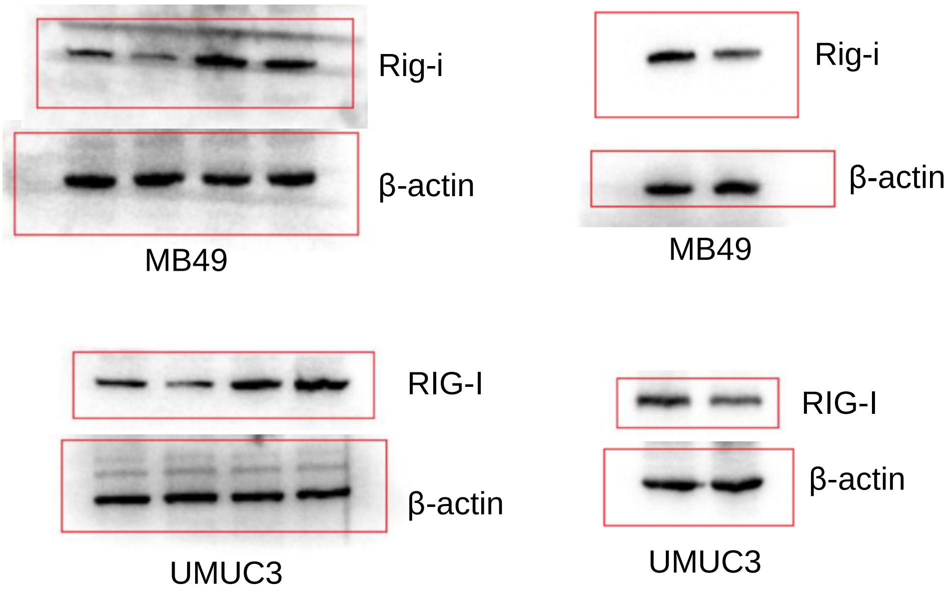

Figure 4L

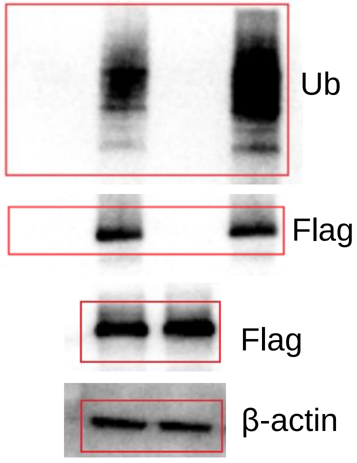

Figure 4M

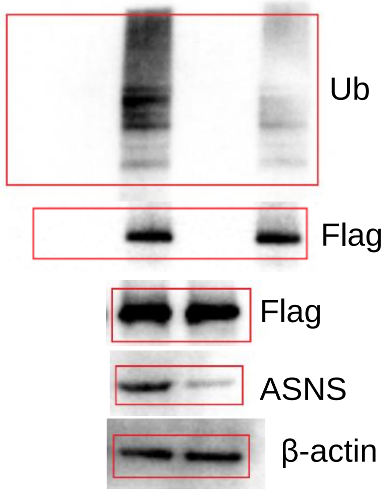

Figure 5A

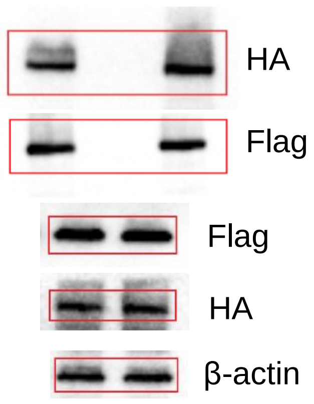

Figure 5B

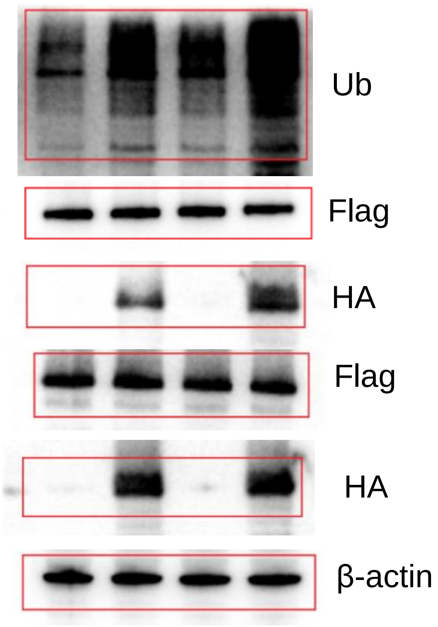

Figure 5C

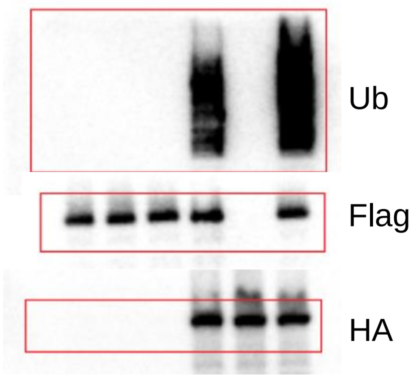

Figure 5F

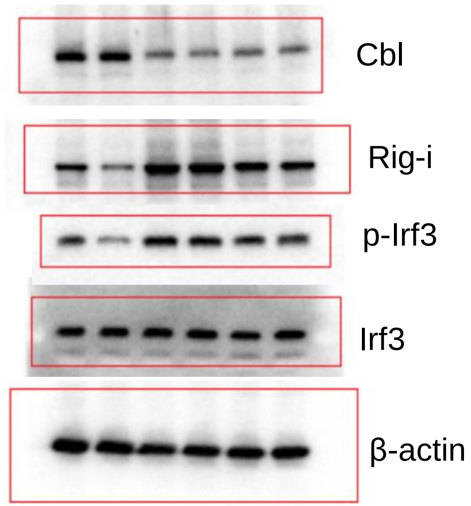

Figure 7B

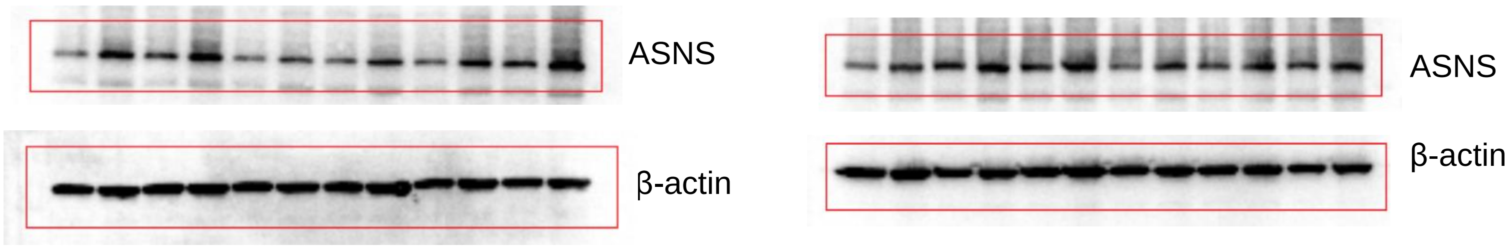

Figure S1A

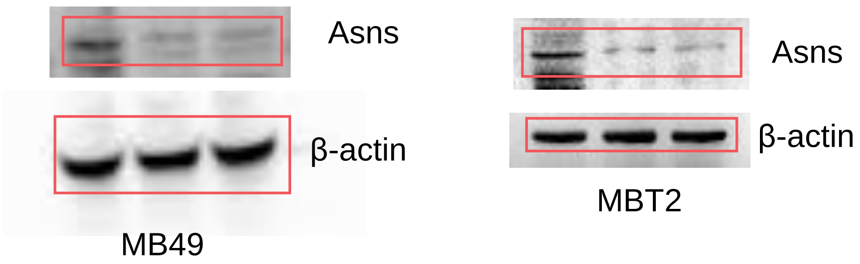

Figure S1B

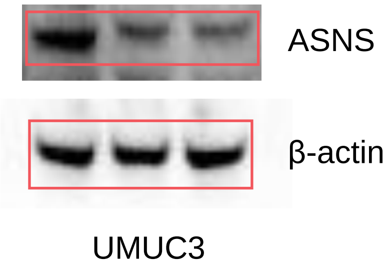

Figure S4G

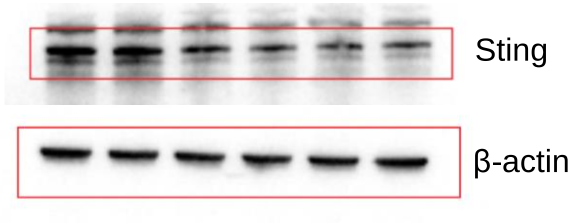

Figure S5C

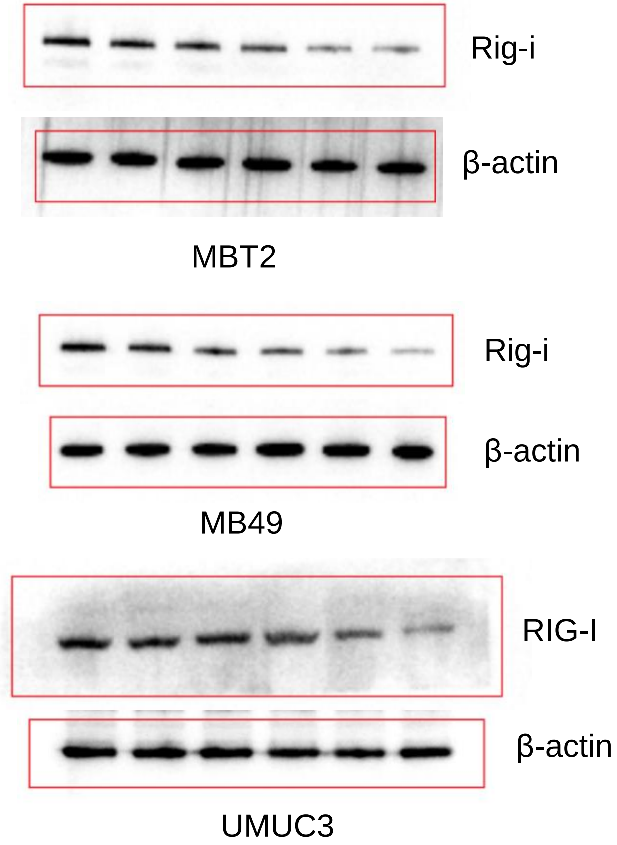

Figure S5D

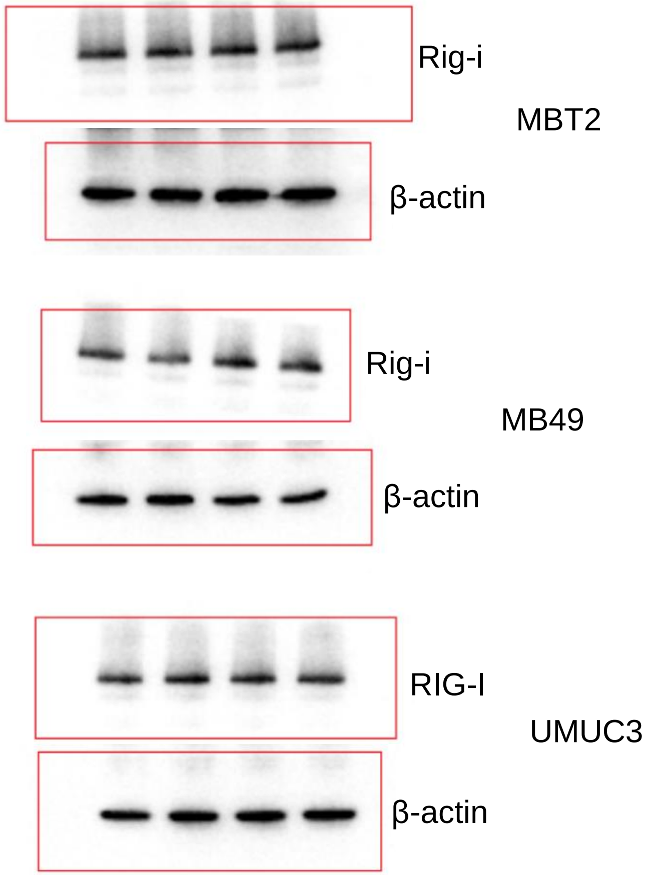

Figure S5B

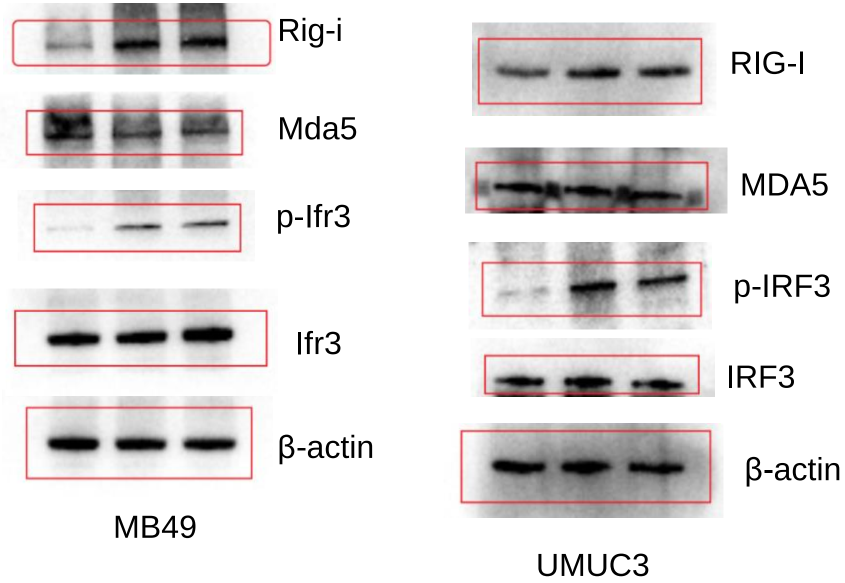

Figure S5E

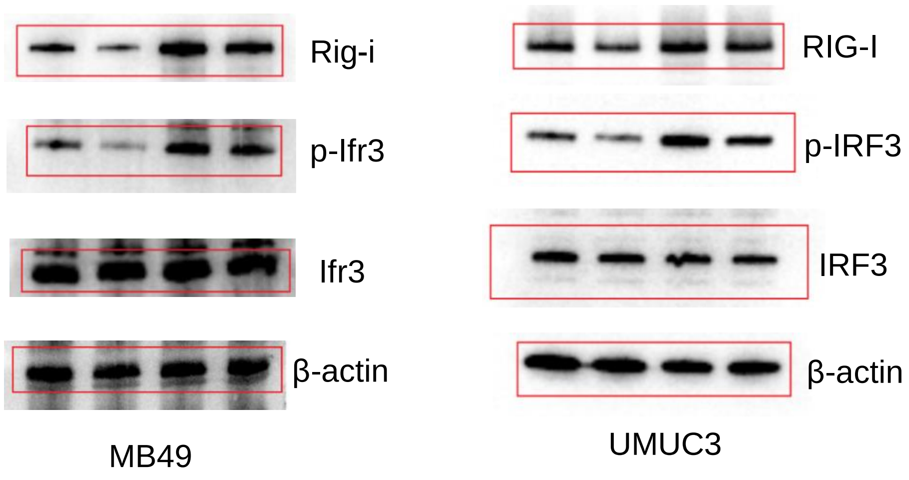

Figure S5F

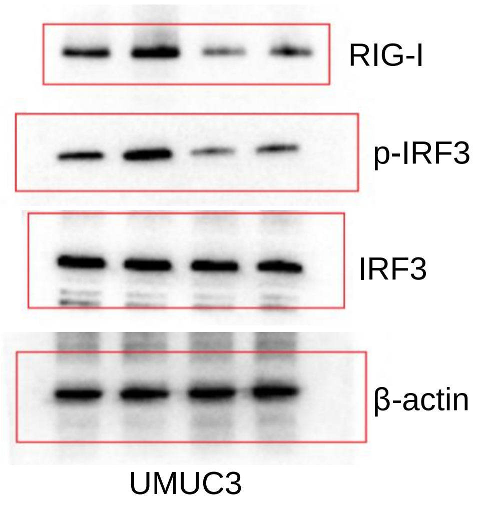

Figure S7A

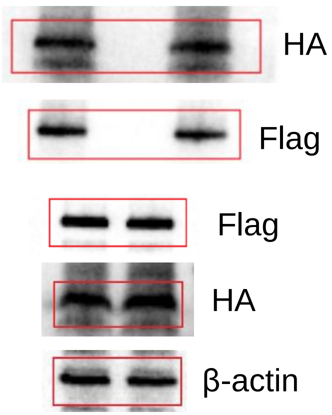

Figure S7B

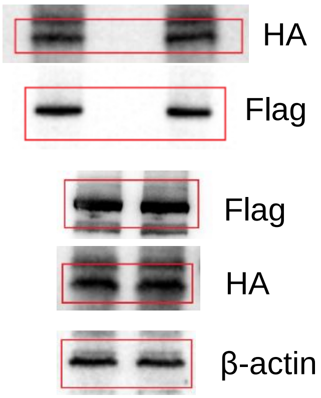

Figure S7C

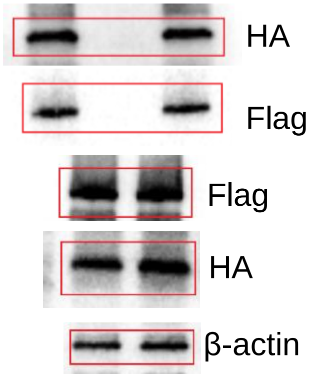

Figure S7D

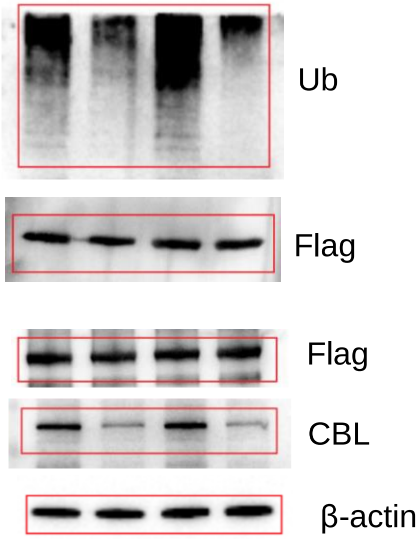

Figure S7E

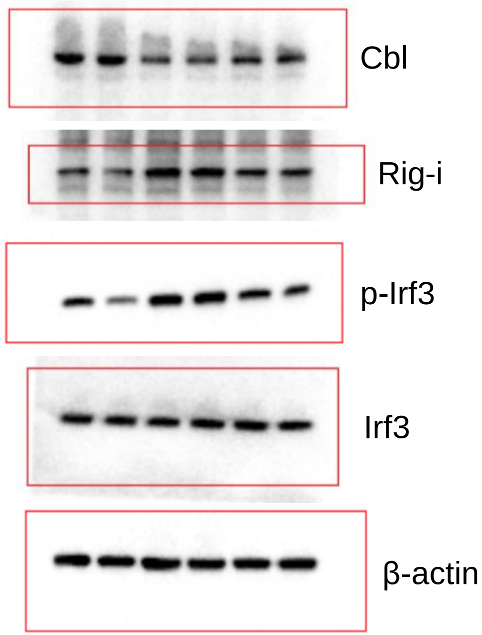

Figure S7F

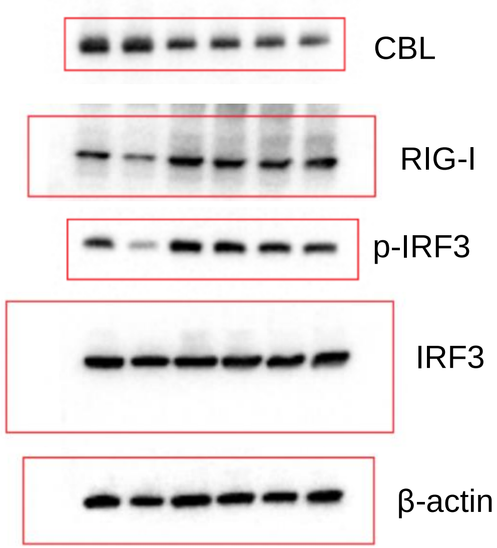

Figure S7I

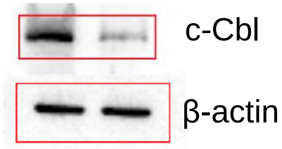

Supplement: Unedited blot and gel images [file jci-135-186648-s081.pdf]
